# Supplementary material for: Medical cost of acute diarrhea in children in ambulatory care
Source: PLoS One. 2022 Dec 16;17(12):e0279239. doi: 10.1371/journal.pone.0279239 (PMC9757569; doi:10.1371/journal.pone.0279239)
Supplement: S1 Table — (DOCX) [file pone.0279239.s001.docx]

**S1 Table. Cases registered as acute diarrhea in the pediatric population under five years of age**

| **Healthcare center** | **Number of consultations (2019)^a^** | **Proportion** | **Sample** |
| --- | --- | --- | --- |
| San Antonio | 286 | 13.30 | 44.2 |
| Comite | 245 | 11.40 | 37.8 |
| Cotocollao | 232 | 10.80 | 35.9 |
| Carcelen Bajo | 142 | 6.60 | 21.9 |
| Puellaro | 135 | 6.30 | 20.9 |
| Colinas del Norte | 122 | 5.70 | 18.9 |
| Condado | 110 | 5.10 | 16.9 |
| Pomasqui | 110 | 5.10 | 16.9 |
| Mena del Hierro | 103 | 4.80 | 15.9 |
| Pisuli | 103 | 4.80 | 15.9 |
| Corazon de Jesus | 90 | 4.20 | 13.9 |
| Centro de Salud 1-E | 77 | 3.60 | 12.0 |
| Jaime Roldos | 71 | 3.30 | 11.0 |
| Atahualpa | 64 | 3.00 | 10.0 |
| San Jose de Minas | 64 | 3.00 | 10.0 |
| La Bota | 58 | 2.70 | 9.0 |
| Chavezpamba | 45 | 2.10 | 7.0 |
| Calacali | 32 | 1.50 | 5.0 |
| Nono | 19 | 0.90 | 3.0 |
| Perucho | 19 | 0.90 | 3.0 |
| Cotocollao Alto | 19 | 0.90 | 3.0 |
| **Total** | **2148** | **100.00** | **332.0** |

^a^Consultations with primary diagnosis of acute diarrhea in under five children
