# Supplementary material for: Medical cost of acute diarrhea in children in ambulatory care
Source: PLoS One. 2022 Dec 16;17(12):e0279239. doi: 10.1371/journal.pone.0279239 (PMC9757569; doi:10.1371/journal.pone.0279239)
Supplement: S2 Table — (DOCX) [file pone.0279239.s002.docx]

**S2 Table. Source and value of unit costs**

| **Medical consultations** | **US$** | **Source** |
| --- | --- | --- |
| **First consult** |  |  |
| General Medicine | 34.31 | Tariff framework of services for the National Health System 2014 current in 2019 |
| Specialist | 34.82 | Tariff framework of services for the National Health System 2014 current in 2019 |
| **Re-consultation** |  |  |
| General Medicine | 22.05 | Tariff framework of services for the National Health System 2014 current in 2019 |
| Specialist | 22.41 | Tariff framework of services for the National Health System 2014 current in 2019 |
| **Medication** |  |  |
| Azithromycin syrup 200 mg/5ml | 2.94 | District Warehouse 2019 expenditure voucher # OUT/2618470 |
| Amoxicillin bottle 250mg/5ml | 4.35 | District Warehouse 2019 expenditure voucher # OUT/2618470 |
| Amoxicillin/clavulanic acid syrup 250/62.5 mg/5ml (100 ml) | 2.64 | District Warehouse 2019 expenditure voucher # OUT/2618470 |
| Clarithromycin syrup 250 mg/5 ml (50 ml) | 6.86 | District Warehouse 2019 expenditure voucher # OUT/2618470 |
| Cefalexin 250 mg/5ml (60 ml) | 2.58 | District Warehouse 2019 expenditure voucher # OUT/2618470 |
| Trimethoprim / Sulfamethoxazole 40/200 mg/5ml (100 ml) | 0.49 | District Warehouse 2019 expenditure voucher # OUT/2618470 |
| Albendazole 100 mg/5ml (20 ml) | 0.41 | District Warehouse 2019 expenditure voucher # OUT/2618470 |
| Metronidazole 250 mg/5ml (120 ml) | 1.75 | District Warehouse 2019 expenditure voucher # OUT/2618470 |
| Ibuprofen 200mg/5ml (120 ml) syrup | 2.24 | District Warehouse 2019 expenditure voucher # OUT/2618470 |
| Paracetamol 100 mg/ml drops (30 ml) | 1.37 | District Warehouse 2019 expenditure voucher # OUT/2618470 |
| Paracetamol 120 mg/5ml syrup (60 ml) | 0.88 | District Warehouse 2019 expenditure voucher # OUT/2618470 |
| Paracetamol 160 mg/5ml syrup (120 ml) | 1.31 | District Warehouse 2019 expenditure voucher # OUT/2618470 |
| Oral rehydration salts | 0.55 | District Warehouse 2019 expenditure voucher # OUT/2618470 |
| Zinc Sulfate oral 5 mg/ml (30 ml) | 4.47 | District Warehouse 2019 expenditure voucher # OUT/2618470 |
| **Laboratory** |  |  |
| Blood count | 5.10 | Tariff framework of services for the National Health System 2014 current in 2019 |
| Urine elemental and microscopic | 5.71 | Tariff framework of services for the National Health System 2014 current in 2019 |
| Gram & fresh drop | 2.66 | Tariff framework of services for the National Health System 2014 current in 2019 |
| Coprological test | 5.47 | Tariff framework of services for the National Health System 2014 current in 2019 |
| White blood cell in stool test | 2.54 | Tariff framework of services for the National Health System 2014 current in 2019 |
| Rotavirus (Stool) | 20.94 | Tariff framework of services for the National Health System 2014 current in 2019 |
| Fecal occult blood test (FOBT) | 3.16 | Tariff framework of services for the National Health System 2014 current in 2019 |

***US$:*** *International dollars 2019*
