# Supplementary material for: Medical cost of acute diarrhea in children in ambulatory care
Source: PLoS One. 2022 Dec 16;17(12):e0279239. doi: 10.1371/journal.pone.0279239 (PMC9757569; doi:10.1371/journal.pone.0279239)
Supplement: Attachment — Submitted filename: PONE-D-22-19266.pdf [file pone.0279239.s003.pdf]

PLOS ONE

Medical Cost of Acute Diarrhea in Children in Ambulatory Care

--Manuscript Draft--

**Manuscript Number:**	PONE-D-22-19266
**Article Type:**	Research Article
**Full Title:**	Medical Cost of Acute Diarrhea in Children in Ambulatory Care
**Short Title:**	Cost of Acute Diarrhea in Children
**Corresponding Author:**	Ruth Jimbo, PhD<br>Pontificia Universidad Catolica del Ecuador<br>Quito, Quito ECUADOR
**Keywords:**	Medical Cost, Diarrhea, Children, Ambulatory Care, Ecuador
**Abstract:**	Objective: The aim of this study was to estimate the direct medical cost per episode and the annual cost for acute diarrhea (AD) in children under five years of age in Ambulatory Health Centers of the Ministry of Public Health (MOPH) of Ecuador.<br>Methods: A cost of illness study with a provider perspective was carried out through a micro-costing of health resources and valuated in international dollars. Medical consultations and laboratory tests were valued using the tariff framework of services for the National Health System and for the prescribed medications, a reported cost registry of pharmacy purchases made in the year of study was used.<br>Results: A total of 332 electronic health records of children under five years of age were included for the analysis. Laboratory tests were performed in 37.95% (126/332), medications were prescribed to 93.67% (311/332) of the children; antimicrobials were prescribed to 37.35% (124/332) of the children, representing an antibiotic prescription rate of 26.51% (88/332) and antiparasitic prescription rate of 10.84% (36/332). The mean cost to the MOPH per child per episode of AD was I$ 45.24 (2019 dollars) (95% CI:43.71 to 46.76).<br>Conclusion: The total estimated cost of AD in children under five years of age for the MOPH in 2019 was about I$6,645,167.88 million (2019 dollars) (95% CI: 6,420,430.77 to 6,868,436.12). A high proportion of the direct medical cost of AD in children under five years of age in outpatient settings is due to the unnecessary use of laboratory tests.
**Order of Authors:**	Xavier Sanchez Choez, PhD.
	Geraldine Leal Rojas, MD.
	Ángel Padilla Hernandez, MD.
	Ruth Jimbo-Sotomayor, PhD.
**Additional Information:**	
**Question**	**Response**
**Financial Disclosure**<br><br>Enter a financial disclosure statement that describes the sources of funding for the work included in this submission. Review the submission guidelines for detailed requirements. View published research articles from *PLOS ONE* for specific examples.<br><br>This statement is required for submission and **will appear in the published article** if the submission is accepted. Please make sure it is accurate.	The authors received no specific funding for this work.

**Unfunded studies**<br>Enter: *The author(s) received no specific funding for this work.*<br><br>**Funded studies**<br>Enter a statement with the following details:<br>• Initials of the authors who received each award<br>• Grant numbers awarded to each author<br>• The full name of each funder<br>• URL of each funder website<br>• Did the sponsors or funders play any role in the study design, data collection and analysis, decision to publish, or preparation of the manuscript?<br>• **NO** - Include this sentence at the end of your statement: *The funders had no role in study design, data collection and analysis, decision to publish, or preparation of the manuscript.*<br>• **YES** - Specify the role(s) played.<br><br>* typeset	
**Competing Interests**<br><br>Use the instructions below to enter a competing interest statement for this submission. On behalf of all authors, disclose any competing interests that could be perceived to bias this work—acknowledging all financial support and any other relevant financial or non-financial competing interests.<br><br>This statement is required for submission and **will appear in the published article** if the submission is accepted. Please make sure it is accurate and that any funding sources listed in your Funding Information later in the submission form are also declared in your Financial Disclosure statement.<br><br>View published research articles from *PLOS ONE* for specific examples.	The authors have declared that no competing interests exist.

**NO authors have competing interests**<br><br>Enter: *The authors have declared that no competing interests exist.*<br><br>**Authors with competing interests**<br><br>Enter competing interest details beginning with this statement:<br><br>*I have read the journal's policy and the authors of this manuscript have the following competing interests: [insert competing interests here]*<br><br>* typeset	
**Ethics Statement**<br><br>Enter an ethics statement for this submission. This statement is required if the study involved:<br><br>• Human participants<br>• Human specimens or tissue<br>• Vertebrate animals or cephalopods<br>• Vertebrate embryos or tissues<br>• Field research<br><br>Write "N/A" if the submission does not require an ethics statement.<br><br>General guidance is provided below. Consult the submission guidelines for detailed instructions. **Make sure that all information entered here is included in the Methods section of the manuscript.**	Ethics approvals for the protocol and the study were granted by Research Ethics on Human Beings–PUCE. The Ethics Committee established the non-need for informed consent for this study.

Powered by Editorial Manager® and ProduXion Manager® from Aries Systems Corporation

**Format for specific study types**<br><br>**Human Subject Research (involving human participants and/or tissue)**<br>• Give the name of the institutional review board or ethics committee that approved the study<br>• Include the approval number and/or a statement indicating approval of this research<br>• Indicate the form of consent obtained (written/oral) or the reason that consent was not obtained (e.g. the data were analyzed anonymously)<br><br>**Animal Research (involving vertebrate animals, embryos or tissues)**<br>• Provide the name of the Institutional Animal Care and Use Committee (IACUC) or other relevant ethics board that reviewed the study protocol, and indicate whether they approved this research or granted a formal waiver of ethical approval<br>• Include an approval number if one was obtained<br>• If the study involved *non-human primates*, add *additional details* about animal welfare and steps taken to ameliorate suffering<br>• If anesthesia, euthanasia, or any kind of animal sacrifice is part of the study, include briefly which substances and/or methods were applied<br><br>**Field Research**<br><br>Include the following details if this study involves the collection of plant, animal, or other materials from a natural setting:<br>• Field permit number<br>• Name of the institution or relevant body that granted permission	
**Data Availability**<br><br>Authors are required to make all data underlying the findings described fully available, without restriction, and from the time of publication. PLOS allows rare exceptions to address legal and ethical concerns. See the PLOS Data Policy and FAQ for detailed information.	Yes - all data are fully available without restriction

Powered by Editorial Manager® and ProduXion Manager® from Aries Systems Corporation

A Data Availability Statement describing where the data can be found is required at submission. Your answers to this question constitute the Data Availability Statement and **will be published in the article**, if accepted.<br><br>**Important:** Stating 'data available on request from the author' is not sufficient. If your data are only available upon request, select 'No' for the first question and explain your exceptional situation in the text box.<br><br>Do the authors confirm that all data underlying the findings described in their manuscript are fully available without restriction?	
**Describe where the data may be found in full sentences. If you are copying our sample text, replace any instances of XXX with the appropriate details.**<br><br>• If the data are **held or will be held in a public repository**, include URLs, accession numbers or DOIs. If this information will only be available after acceptance, indicate this by ticking the box below. For example: *All XXX files are available from the XXX database (accession number(s) XXX, XXX.).*<br>• If the data are all contained **within the manuscript and/or Supporting Information files**, enter the following: *All relevant data are within the manuscript and its Supporting Information files.*<br>• If neither of these applies but you are able to provide **details of access elsewhere**, with or without limitations, please do so. For example:<br><br>*Data cannot be shared publicly because of [XXX]. Data are available from the XXX Institutional Data Access / Ethics Committee (contact via XXX) for researchers who meet the criteria for access to confidential data.*<br><br>*The data underlying the results presented in the study are available from (include the name of the third party*	All relevant data are within the manuscript and its Supporting Information files.

and contact information or URL).<br>• This text is appropriate if the data are owned by a third party and authors do not have permission to share the data.<br><br>* typeset	
Additional data availability information:	Tick here if the URLs/accession numbers/DOIs will be available only after acceptance of the manuscript for publication so that we can ensure their inclusion before publication.

Powered by Editorial Manager® and ProduXion Manager® from Aries Systems Corporation

Manuscript

Click here to access/download;Manuscript;Manuscript-Medical Cost Diarrhea.pdf

Title

Medical Cost of Acute Diarrhea in Children in Ambulatory Care

Authors:

Xavier Sánchez[1], Gerardine Leal[2], Angel Padilla[2], Ruth Jimbo-Sotomayor[1]

[1] Centro de Investigación en Salud para América Latina (CISeAL), Facultad de Medicina, Pontificia Universidad Católica del Ecuador (PUCE)

[2] Postgrado de Medicina Familiar y Comunitaria, Pontificia Universidad Católica del Ecuador (PUCE)

Abstract:

Objective: The aim of this study was to estimate the direct medical cost per episode and the annual cost for acute diarrhea (AD) in children under five years of age in Ambulatory Health Centers of the Ministry of Public Health (MOPH) of Ecuador.

Methods: A cost of illness study with a provider perspective was carried out through a micro-costing of health resources and valuated in international dollars. Medical consultations and laboratory tests were valued using the tariff framework of services for the National Health System and for the prescribed medications, a reported cost registry of pharmacy purchases made in the year of study was used.

Results: A total of 332 electronic health records of children under five years of age were included for the analysis. Laboratory tests were performed in 37.95% (126/332), medications were prescribed to 93.67% (311/332) of the children; antimicrobials were prescribed to 37.35% (124/332) of the children, representing an antibiotic prescription rate of 26.51% (88/332) and antiparasitic prescription rate of 10.84% (36/332). The mean cost to the MOPH per child per episode of AD was I$ 45.24 (2019 dollars) (95% CI:43.71 to 46.76).

Conclusion: The total estimated cost of AD in children under five years of age for the MOPH in 2019 was about I$6,645,167.88 million (2019 dollars) (95% CI: 6,420,430.77 to 6,868,436.12). A high proportion of the direct medical cost of AD in children under five years of age in outpatient settings is due to the unnecessary use of laboratory tests.

Introduction

Diarrheal disease is one of the leading causes of death in children in developing countries [1]. Acute diarrhea (AD) is defined as the passage of three or more loose or liquid stools per day [2], it primarily occurs in children during the first five years after birth, and particularly in the second half-year and in little children [3]. It is estimated that 2.5 billion cases of diarrheal disease occur each year in children under five years of age, and an average of more than 1,400 children die each day [4]. Evidence shows that AD is common in places with poor access to health care, safe water and sanitation, which is often observed in low and middle-income countries [5].

The most common cause of AD is gastrointestinal infections caused by virus, bacteria and rarely parasitic. Rotavirus is responsible for 60-70%% of all diarrheal diseases; however, E. coli, Campylobacter, Yersinia and Salmonella spp. are common bacterial causes of AD [6]. The diagnosis of AD is mainly based on the medical interview, which provide accurate information about the duration of diarrhea and the characteristics of the accompanying symptoms, such as

abdominal pain, vomiting and fever. Healthy children without any comorbidity do not require specific diagnosis tests in cases of AD since the results of laboratory and microbiological tests do not change the treatment of the episode; so, testing should be kept to a minimum and reserved for patients with severe illness [7].

Most episodes of AD are self-limiting, and prevention of dehydration is the main goal of diarrhea treatment. Medications such as antibiotics, antiparasitic drugs and antidiarrheal agents are not necessary and can be harmful for infants or children with diarrhea [8,9]. Rarely, antibiotics may be used in cases of bacterial infection when a specific cause of the diarrhea has been found or is strongly suspected, particularly after a recent travel [10]. However, evidence shows that the most commonly reported inappropriate measures used in AD are the unwarranted prescription of antimicrobial, antidiarrheal and antimotility drugs, especially in low- and middle-income countries [11].

Unnecessary use of resources in the treatment of AD leads to inefficient use of scarce healthcare resources. It is estimated that a significant proportion of medical tests and drug prescribing is unnecessary around the world; this is of particular concern because of the potential financial effect of excessive resource utilization on healthcare systems [12]. To date, no research has attempted to estimate this cost burden on Ecuador's healthcare system, even when AD is a leading cause of morbidity in children [13]. Showing the importance of this problem to policy makers and clinicians would provide information to aid budgetary decision making and understanding of the need for cost containment interventions.

Materials and Methods

Objective

This study aimed to estimate the direct cost per episode and the annual cost of AD in children under five years of age in the Ambulatory Health Centers of the Ministry of Public Health (MOPH) of Ecuador in 2019.

Setting

Ecuador's health system is made up of two sectors, public and private. The MOPH is the governing entity of health in the country, offering health care services to the entire Ecuadorian population, its financing comes from state contributions and within the public health system it has a coverage of about 62%; the rest of the population is covered by a public subsystem such as social security institutions like the Ecuadorian Institute of Social Security (IESS), Institute of Security of the Armed Forces (ISSFA) and Institute of Social Security of the National Police (ISSPOL)] or the private system for a population group that has the capacity to pay [14–16].

District 17D03 of the city of Quito belongs to the public health network of Ecuador. This district has a total assigned population of 430,000 inhabitants and almost 36% is a pediatric population (0-18 years). The district is distributed in 21 ambulatory medical care centers, These health centers are distributed in rural and urban areas of the city and offer health services to 85% of the population [16,17].

Sample

A simple probabilistic type sampling was performed. To calculate the sample, the total number of cases registered by AD in the pediatric population under five reported by the MOPH in the district in 2019 was used. We considered as acute diarrhea diagnosis as any diagnosis registered according to the ICD-10 classification, as the following: **A00:** Cholera, **A01:** Typhoid and paratyphoid fevers, **A02:** Other salmonella infections, **A03:** Shigellosis, **A04:** Other bacterial intestinal infections, **A05** Other bacterial foodborne intoxication not elsewhere classified, **A06:** Amoebiasis, **A07:** Other protozoal intestinal diseases, **A08:** Viral and other specified intestinal infections and **A09:** Other gastroenteritis and colitis of infectious and unspecified origin.

The following formula was applied to calculate the sample for a finite universe, $n = N*Z^2*p*q / d^2*(N-1) + Z^2*p*q$.

Where N is the population size, Z is the confidence level (95%), p is the probability of success, or expected proportion (50%), q is the probability of failure (50%) and d is precision (5% of maximum admissible error in terms of proportion). We decided to increase the sample by 10% considering possible losses. The subsequently studied sample comprised 332 Electronic Health Records (EHR).

Identification of resource use and data collection

Perspective

A provider perspective was used that included resources provided by the MOPH during the period between the initial consultation and resolution of the illness up to a maximum of five weeks. This included medical care, laboratory tests, and medication as primary resources.

Data source

The data source for the resources used and data collection included in this study were the Electronic Health Records (EHR) of the patients from one Health Center of the Ministry of Public Health in the District 17D03 in Ecuador during 2019 with AD as a primary diagnosis. This district has been using the EHR since 2010. Physicians enter information directly into the EHR on a computer during the outpatient appointment. Information was manually extracted from the EHR by two reviewers. Tests ordered and medication administered were verified against laboratory and pharmacy records.

Valuation of resources

All resources were valued in dollars at 2019 prices. To make the monetary values internationally comparable, they were converted to international dollars (I$) using the purchasing power parity (PPP) conversion factor for Ecuador [18]. Resource use was valued using recognized sources of costs, unit costs and their source are available in Supporting Information S1, using the service tariff framework for the National Health System [19] and for the prescribed medication a record of reported costs of pharmacy purchases made in the study year was used. No discount was necessary since the period was one year.

Estimating the annual cost to the MOPH

To estimate the annual cost of AD in 2019 for the MOPH in children under five years of age, the estimated cost per episode was combined with the total number of cases of AD treated by the MOPH at the first level of health for that population. The annual cost to the population was calculated as follows: *Annual cost 2019 = (cost per episode per child per year 2019) × (total number of episodes per year 2019).* The number of episodes per year 2019 is the number of children

under five years of age consulting with an AD in a primary health center of the MOPH in 2019, according to ICD-10 codes taken from the official of national statistics(13). All analyses were carried out using SPSS v25 software.

Results

Participant characteristics

Three hundred and thirty-two eligible EHR were randomly selected and proofed of suitability for inclusion in the cost-of-illness study. The general characteristics of our sample are described in Table 1.

Table 1. Characteristics of the sample

Sample	n (%)
**Gender of Professionals**	
Female	59 (64.84)
Male	32 (35.16)
**Age of Professionals**	
Mean (SD)	40.51 (13.41)
**Health Professionals**	
Rural Doctor	5 (5.49)
General Practitioner	57 (62.63)
Family Medicine Physician	26 (28.57)
Pediatrician	3 (3.29)
**Health professional consultations**	
Rural Doctor consultations	57 (17.16)
General Practitioner consultations	152 (45.78)
Family Medicine Physician consultations	39 (11.74)
Pediatrician consultations	84 (25.30)
**Gender of patients**	
Female	141 (42.46)
Male	191 (57.53)
**Age of patients**	
Mean (SD)	1.85 (1.29)
**Comorbidity**	
Any comorbidity	34 (10.24)
No comorbidity	298 (89.75)
**Total of EHR**	**332 (100)**

SD: *Standard Deviation,* ***EHR:*** *Electronic health records*

A total of 91 healthcare professionals treated all the cases coded as AD, the female gender was the most frequent (64.84% vs. 35.16%) and the mean age of the professionals was 40.5. Two types of health care professionals were included, general physicians (Rural Doctor and General Practitioner) and specialists (Family Medicine Physician and Pediatrician), general physicians were the most frequent health professionals (68.12% vs. 31.88%).

Of the total number of AD consultations, general practitioner consultations were more frequent with 45.78% (152/332) followed by pediatrician consultations with 25.30% (84/332).

Male gender of children was more common (57.53% vs. 42.46%), the mean age was 1.29; and most of the registered children did not have comorbidities (89.75% vs. 10.24%).

The ICD-10 diagnostic code most frequently assigned by physicians was A09 (Other gastroenteritis and colitis of infectious and unspecified origin) in 98.19% (326/332) of the cases (Table 2).

Table 2. Diagnosis of acute diarrhea cases by each type of health professional

**Health professional**	**ICD-10 diagnosis n (%)**				**Total**
	**A06.9**	**A07.1**	**A08.4**	**A09**	
Rural Doctor	0 (0)	0 (0)	0 (0)	57 (17.49)	57
General Practitioner	1 (25)	1 (100)	0 (0)	150 (46.01)	152
Family Medicine Physician	0 (0)	0 (0)	0 (0)	39 (11.96)	39
Pediatrician	3 75)	0 (0)	1 (100)	80 (24.54)	84
**Total**	**4 (100)**	**1(100)**	**1 (100)**	**326 (100)**	**332**

A06.9: *Amoebiasis, unspecified,* ***A07.1:*** *Giardiasis [lambliasis],* ***A08.4:*** *Viral intestinal infection, unspecified,* ***A09:*** *Other gastroenteritis and colitis of infectious and unspecified origin*

Resource use

The total resource use in the sample by item, is given in Table 3. A total of 1546 resources were used in 332 children. All children had one primary care contact, and the re-consultation rate was 9.94% (33/332); out of these, just one child needed two re-consultations.

Table 3. Total resource use and cost per episode per participant

Direct sanitary resources	Number of patients	Units dispensed	Mean Cost per child (95%IC)
**First consultations**	**332**	**332**	**34.49 (34.40 – 34.55)**
**Re-consultations**	**33**	**34**	**22.88 (21.53 – 24.24)**
**Laboratory tests**	**126**	**320**	**15.98 (14.20 – 17.76)**
Blood count	38	38	5.10 (5.10 – 5.10)
Urinalysis	22	22	5.69 (5.69 – 5.69)
Gram stain & fresh drop	3	3	2.75 (2.75 – 2.75)
Coprological test	123	123	5.29 (5.29 – 5.29)
WBC in stool test	80	80	2.55 (2.55 – 2.55)
Rotavirus	36	36	21.37 (21.37 – 21.37)
FOBT	18	18	3.14 (3.14 – 3.14)
**Prescriptions**	**311**	**860**	**2.51 (2.27 – 2.76)**
Antibiotics	88	96	1.75 (1.27 – 2.22)
Antiparasitic	36	57	1.84 (1.65 – 2.02)
Anti-inflammatory	135	136	1.31 (1.25 – 1.37)
Oral rehydration salts	242	553	1.25 (1.18 – 1.33)
Zinc Sulfate	12	18	6.10 (4.59 – 7.61)
**Total cost**	**332**	**1546**	**45.24 (43.71 – 46.76)**

IC: *Confident interval,* ***WBC:*** *White blood cell,* ***FOBT****: Fecal occult blood test* ***Note:*** *Cost presented as International Dollars 2019*

Laboratory tests were performed in 37.95% (126/332) of the children, representing 320 units of laboratory tests dispensed, of which 38.44% (123/320) were coprological tests, 25% (80/320) stool white blood cell tests, 11.25% (36/320) rotavirus tests, 11.88% (38/320) blood count, and 6.88% (22/320) urinalysis (Table 3).

Medications were prescribed to 93.67% (311/332) of the children. Oral rehydration salts were the most prescribed medication, with 77.81% (242/311), followed by anti-inflammatory drugs with 43.41% (135/311). Antibiotics were prescribed to 88 children, representing an antibiotic prescription rate of 26.51% (88/332) and antiparasitic were prescribed to 36 children, indicating a prescription rate of 10.84% (36/332). Zinc sulfate was prescribed to only 3.61% (12/332) (Table 3).

A total of 860 units of medications were dispensed, of these oral rehydration salts representing 60.30% (553/860) of units used, anti-inflammatory drugs 17.79% (136/860), antibiotics 11.16% (96/860), antiparasitic 6.63% (57/860) and zinc sulfate 2.09% (18/860) (Table 3).

Medical contact accounts for 23.67% (366/1546) of total resource use, prescriptions account for 55.63% (860/1546) and laboratory tests account for 20.70% (320/1546) (Table 4).

Table 4. Total resource use and overall cost per direct sanitary resources

**Direct sanitary resources**	**Number of patients**	**Units dispensed**	**% Resource use**	**Overall Cost**	**% Overall Cost**
First consultations	332	332	21.47	1,1462.98	76.38
Re-consultations	33	34	2.20	755.29	5.03
Laboratory tests	126	320	20.70	2,007.74	13.38
Prescriptions	311	860	55.63	782.14	5.21
**Total cost**	**332**	**1546**	**100.00**	**15,008.15**	**100.00**

Note: *Cost presented as International Dollars 2019*

Cost per episode and overall cost

The mean cost per child per resource used is given in Table 3. The mean cost to the MOPH per child per episode of AD was I$ 45.24 (2019 dollars) (95% CI:43.71, 46.76), the mean cost of

laboratory tests was I$ 15.98 (95% CI: 14.20, 17.76) (2019 dollars) and the mean cost of drugs prescription was I$ 2.51 (95% CI: 2.27, 2.76) (2019 dollars).

The overall cost for the whole sample is given in Table 4. The total cost of AD for the whole sample was I$ 15,008.15 (95% CI: 14,511.72, 15,524.32) (2019 dollars). The first consultation accounted for 76.38% of the total cost for the sample and the subsequent consultations accounted for 5.03%. Laboratory tests accounted for 13.38% (I$ 2,007.74) (2019 dollars) and prescriptions accounted for 5.21% (I$ 782.14) (2019 dollars) of the overall cost.

The cost by type of laboratory test is shown in Table 5. Rotavirus tests accounted for 38.32% of the overall cost of laboratory tests, follow by coprological test with 32.41% and white blood cells in stool test with 10.16%.

The cost by type of prescription is shown in Table 6. Oral rehydration salts accounted for 38.82% of the overall cost of prescription, followed by any type of antimicrobial drug (antibiotic/antiparasitic) with 28.13%, analgesics/anti-inflammatory drugs with 22.73%, and zinc sulfate with 10.29%.

Table 5. Cost by type of laboratory test

Type of laboratory test	Units	Total Cost	%
Blood count	38	193.8	9.65
Urinalysis	22	125.18	6.23
Gram stain & fresh drop	3	8.25	0.41
Coprological test	123	650.67	32.41
WBC in stool test	80	204	10.16
Rotavirus	36	769.32	38.32
FOBT	18	56.52	2.82
**Total**	**320**	**2,007.74**	**100**

WBC: *White blood cell,* ***FOBT****: Fecal occult blood test.* ***Note:*** *Cost presented as International Dollars 2019*

Table 6. Cost by type of medication

Type of medication	Units	Total Cost	%
Amoxicillin	14	59.24	7.57
Azithromycin	9	20.94	2.68
Cefalexin	3	7.76	0.99
Clarithromycin	5	34.31	4.39
Trimethoprim / Sulfamethoxazole	65	31.86	4.07
Metronidazole	32	55.84	7.14
Albendazole	25	10.29	1.32
Ibuprofen	9	21.43	2.74
Paracetamol	127	156.37	19.99
Oral rehydration salts	553	303.63	38.82
Zinc Sulfate	18	80.47	10.29
**Total**	**860**	**782.14**	**100**

Note: *Cost presented as International Dollars 2019*

Annual Cost to MOPH

We combined the cost per episode results with data on prevalence. The number of consultations for AD in children under five years of age in the ambulatory health centers of the MOPH, according to the National Direction of Statistics and Analysis of the Health Information (DNEAIS), in 2019 was 146,887 (20). Thus, the annual cost to the MOPH was at least I$ 6,645,167.88 million (2019 dollars) (95% CI: 6,420,430.77 to 6,868,436.12).

Sensitivity analysis

The results coming from the cost of illness study may be conservative; the effect of the proportion of laboratory test is explored here. Children otherwise healthy with AD in ambulatory settings do not require a specific diagnostic work-up, as the results of laboratory investigations are unlikely to change their management [10,21]. According to the Integrated Management of Childhood Illness (IMCI)(22), among other components, improve the performance of health personnel in the care of

children under five, it is not necessary to request laboratory tests in outpatient care. However, we found a proportion of laboratory tests ordered of around 13%, if we consider that physicians comply with the recommendations for care according to IMCI, the estimated population cost would be eight hundred thousand dollars lower. Other variables in the study do not greatly affect the results as we found a relative low cost of them.

Discussion

This is the first study to measure AD management costs in children under five years of age in Ecuador. Our data provide reliable statistics on resource use and costs from a payer perspective. The cost was estimated from medical records with diagnoses coded according to ICD-10, applying a micro-cost methodology by involving the direct identification and valuation of the resources consumed treating each patient, which improves the accuracy of cost estimation [23].

Medical appointments represent 23.67% of the resources used per AD episode. All children had at least an initial consultation and the rate of re-consultation was almost 10%, which is less than that reported in literature [24]. Laboratory tests were ordered in almost 38% of the children, accounting around the 20% of the resources used.

Medication prescription was the most frequently used resource in the treatment of AD cases. It accounted for 55.63% of all resource units administered. Oral rehydration salts, anti-inflammatory drugs and antibiotics were the most prescribed drugs, 72.89%, 40.66%, and 26.51% respectively; these results are similar to other studies [25–27]. Regardless of this, it is important to mention that the use of resources in medical care could vary according to their availability.

Numerous studies have reported the economic burden of childhood diarrhea in different settings; however, empirical data on the cost of diarrheal illness is sparse. We found that the average cost

of AD per episode in children under five years of age in Ecuador was I$ 45.24 (2019 dollars). Our results are similar to those of some countries reported by Rheingans et al. [28] for Latin American and Caribbean countries, where total direct medical cost in outpatient settings was I$ 18.45 in Argentina, I$ 15.03 in Brazil, 47.25 I$ in Chile, I$ 30.26 in Honduras, I$ 33.58 in Mexico, I$ 39.50 in Panamá and I$ 30.96 in Venezuela (monetary change to 2019). Nevertheless, the proportion of laboratory and medication costs were higher in Rheingans' study, between 50% and 80%, compared to our data, which was less than 20%. Alternatively, a systematic review of the cost of childhood diarrhea in 137 low- and middle-income countries (LMICs) estimated an averaged (weighted) I$52.16 per outpatient episode of AD [29], and the direct medical cost represented almost 80% of the total cost, I$41.21 (2020 dollars), these findings are more comparable with our results.

Considerable variation is found in the cost estimates reported in the literature, the variation that can be attributed to methodological differences between studies, in particular variation in the perspective adopted by the studies and the categorization of the costs [30–32]. Additionally, the direct cost associated with treatment could vary according to the capabilities and resources available in different health units, even in the same country, leading to some heterogeneity in the estimated costs of the studies.

Cost-of-illness studies can estimate cost based on epidemiological data, through incidence-based or prevalence-based approaches [33], the latter is useful for identifying points for cost containment, as this approach provides information on each cost component [34,35].

The total estimated cost of AD in children under five years of age for MOPH in 2019 was about I$6,645,167.88 million (2019 dollars) (95% CI: 6,420,430.77 to 6,868,436.12). If laboratory tests had been minimized and antibiotic use cut by at least half, our annual cost estimate would have

been reduced by $1 million. Therefore, an intervention that promotes rational use of these resources would be cost a saving.

The Integrated Management of Childhood Illness (IMCI) strategy was designed in 1996 by the World Health Organization (WHO) and the United Nations Children's Fund (UNICEF) to improve the quality of care for children in primary care services and was adopted by Ecuador [36]. According to this strategy, in patients under five years of age with AD, it is not necessary to request laboratory tests, and the use of antibiotics is justified in few circumstances; therefore, our findings show that there may be an overuse of these resources and lack of adherence to IMCI recommendations, a situation that has been reported in other studies [37,38]; however, the evaluation of the strategy and its implementation is beyond our research objectives.

A limitation of this study is that it focuses only on the population subsidized by the MOPH; however, the MOPH health subsystem covers most of the country's population [14,39]; therefore, the results can be generalized to other public health subsystems. Another limitation is the perspective used in the study, we did not include patient time costs or other associated resource costs, this perspective is narrower and does not include patients' out-of-pocket costs as they are not borne by payers; therefore, the cost of illness may be underestimated. A study strength is that the AD cases were selected from the medical records of different health centers, according to ICD-10, which allowed us to obtain data according to their own diagnoses and thus determine the use of resources for such care; moreover, the study shows relevant data for decision making stakeholders to consider cost containment measures for efficient use of medical resources.

Conclusion:

A high proportion of the direct medical cost of AD in children under five years of age in outpatient settings is due to the unnecessary use of laboratory tests. Factors that may influence the overuse of this resource in primary care need to be explored.

Acknowledgements:

None

References:

1. Hug L, Sharrow D, Danzhen Y, on behalf of the United Nations Inter-agency Group for Child Mortality Estimation (UN IGME). Levels & Trends in Child Mortality: Report 2019. UNICEF [Internet]. 2019;52. Available from: https://www.unicef.org/media/60561/file/UN-IGME-child-mortality-report-2019.pdf
2. Drancourt M. Acute Diarrhea. In: Infectious Diseases [Internet]. Elsevier; 2017. p. 335-340.e2. Available from: https://linkinghub.elsevier.com/retrieve/pii/B9780702062858000381
3. Radlovic N, Lekovic Z, Vuletic B, Radlovic V, Simic D. Acute diarrhea in children. Srp Arh Celok Lek [Internet]. 2015;143(11–12):755–62. Available from: http://www.doiserbia.nb.rs/Article.aspx?ID=0370-81791512755R
4. Pinzón-Rondón ÁM, Zárate-Ardila C, Hoyos-Martínez A, Ruiz-Sternberg ÁM, Vélez-van-Meerbeke A. Country characteristics and acute diarrhea in children from developing nations: a multilevel study. BMC Public Health [Internet]. 2015 Dec 21;15(1):811. Available from: http://bmcpublichealth.biomedcentral.com/articles/10.1186/s12889-015-

2120-8

5. Troeger C, Forouzanfar M, Rao PC, Khalil I, Brown A, Reiner RC, et al. Estimates of global, regional, and national morbidity, mortality, and aetiologies of diarrhoeal diseases: a systematic analysis for the Global Burden of Disease Study 2015. Lancet Infect Dis [Internet]. 2017 Sep;17(9):909–48. Available from: https://linkinghub.elsevier.com/retrieve/pii/S1473309917302761
6. Cheng AC, McDonald JR, Thielman NM. Infectious Diarrhea in Developed and Developing Countries. J Clin Gastroenterol [Internet]. 2005 Oct;39(9):757–73. Available from: http://journals.lww.com/00004836-200510000-00002
7. Aranda-Michel J, Giannella RA. Acute diarrhea: a practical review. Am J Med [Internet]. 1999 Jun;106(6):670–6. Available from: https://linkinghub.elsevier.com/retrieve/pii/S000293439900128X
8. Florez ID, Veroniki A-A, Al Khalifah R, Yepes-Nuñez JJ, Sierra JM, Vernooij RWM, et al. Comparative effectiveness and safety of interventions for acute diarrhea and gastroenteritis in children: A systematic review and network meta-analysis. van Wouwe JP, editor. PLoS One [Internet]. 2018 Dec 5;13(12):e0207701. Available from: https://dx.plos.org/10.1371/journal.pone.0207701
9. Lo Vecchio A, Dias JA, Berkley JA, Boey C, Cohen MB, Cruchet S, et al. Comparison of Recommendations in Clinical Practice Guidelines for Acute Gastroenteritis in Children. J Pediatr Gastroenterol Nutr [Internet]. 2016 Aug;63(2):226–35. Available from: https://journals.lww.com/00005176-201608000-00011
10. Guarino A, Lo Vecchio A, Dias JA, Berkley JA, Boey C, Bruzzese D, et al. Universal Recommendations for the Management of Acute Diarrhea in Nonmalnourished Children. J Pediatr Gastroenterol Nutr [Internet]. 2018 Nov;67(5):586–93. Available from:

https://journals.lww.com/00005176-201811000-00011

11. Carter E, Bryce J, Perin J, Newby H. Harmful practices in the management of childhood diarrhea in low- and middle-income countries: a systematic review. BMC Public Health [Internet]. 2015 Dec 18;15(1):788. Available from: http://bmcpublichealth.biomedcentral.com/articles/10.1186/s12889-015-2127-1

12. Brownlee S, Chalkidou K, Doust J, Elshaug AG, Glasziou P, Heath I, et al. Evidence for overuse of medical services around the world. Lancet [Internet]. 2017 Jul;390(10090):156–68. Available from: https://linkinghub.elsevier.com/retrieve/pii/S0140673616325855

13. Ministerio de Salud Pública del Ecuador. Información Estadística y Geográfica de Salud [Internet]. 2017 [cited 2020 Jun 7]. Available from: https://www.salud.gob.ec/informacion-estadistica-de-produccion-de-salud/#

14. Ministerio de Salud Pública del Ecuador. En 2018: se evidenció un Sistema Nacional de Salud fortalecido – Ministerio de Salud Pública [Internet]. 2018 [cited 2020 Jun 2]. Available from: https://www.salud.gob.ec/en-2018-se-evidencio-un-sistema-nacional-de-salud-fortalecido/

15. Lucio R, Villacrés N, Henríquez R. Sistema de salud de Ecuador. Salud Publica Mex [Internet]. 2011 [cited 2015 Jan 15];53(1):177–87. Available from: http://www.scielo.org.mx/scielo.php?pid=S0036-36342011000800013&script=sci_arttext

16. Instituto Nacional de Estadísticas y Censos (INEC-Ecuador). Encuesta de Condiciones de Vida. ECV [Internet]. 2015. Available from: https://www.ecuadorencifras.gob.ec/encuesta-de-condiciones-de-vida-ecv

17. Ministerio de Salud Pública del Ecuador. Manual del Modelo de Atencion Integral del Sistema Nacional de Salud Familiar Comunitario e Intercultural (MAIS-FCI) [Internet].

2012 [cited 2019 Jun 28]. Available from: http://instituciones.msp.gob.ec/somossalud/images/documentos/guia/Manual_MAIS-MSP12.12.12.pdf

18. International Monetary Fund. World Economic Outlook Database October 2017 [Internet]. [cited 2020 Jun 2]. Available from: https://www.imf.org/external/pubs/ft/weo/2017/02/weodata/index.aspx

19. Ministerio de Salud Pública del Ecuador. Tarifario de Prestaciones para el Sistema Nacional de Salud [Internet]. 2014. Available from: https://aplicaciones.msp.gob.ec/salud/archivosdigitales/documentosDirecciones/dnn/archivos/TARIFARIO.pdf

20. Dirección Nacional de Estadística y Análisis de Información de Salud – Ministerio de Salud Pública. Registro Diario Automatizado de Consultas y Atenciones Ambulatorias (RDACAA 1.6 y RDACAA 2.0) y Plataforma de registro en atenciones de salud (PRAS) [Internet]. 2019 [cited 2022 Apr 21]. Available from: https://www.salud.gob.ec/direccion-nacional-de-estadistica-y-analisis-de-informacion-de-salud/

21. Florez ID, Niño-Serna LF, Beltrán-Arroyave CP. Acute Infectious Diarrhea and Gastroenteritis in Children. Curr Infect Dis Rep [Internet]. 2020 Feb 28;22(2):4. Available from: http://link.springer.com/10.1007/s11908-020-0713-6

22. Ministerio de Salud Pública del Ecudor - Dirección Nacional de Normatización. Atención Integrada a Enfermedades Prevalentes de la Infancia (AIEPI) Clínico. Cuadro de Procedimientos. Actualización 2017 [Internet]. Quito; 2017. 92 p. Available from: http://instituciones.msp.gob.ec/images/Documentos/AIEPI_6feb_2019_web.pdf

23. Xu X, Grossetta Nardini HK, Ruger JP. Micro-costing studies in the health and medical literature: protocol for a systematic review. Syst Rev [Internet]. 2014 Dec 21;3(1):47.

Available from: https://systematicreviewsjournal.biomedcentral.com/articles/10.1186/2046-4053-3-47

24. Rautenberg TA. Evaluating the cost utility of racecadotril for the treatment of acute watery diarrhea in children: the RAWD model. Clin Outcomes Res [Internet]. 2012 Apr;109. Available from: http://www.dovepress.com/evaluating-the-cost-utility-of-racecadotril-for-the-treatment-of-acute-peer-reviewed-article-CEOR

25. Okubo Y, Miyairi I, Michihata N, Morisaki N, Kinoshita N, Urayama KY, et al. Recent Prescription Patterns for Children With Acute Infectious Diarrhea. J Pediatr Gastroenterol Nutr [Internet]. 2019 Jan;68(1):13–6. Available from: https://journals.lww.com/00005176-201901000-00004

26. Efunshile AM, Ezeanosike O, Nwangwu CC, König B, Jokelainen P, Robertson LJ. Apparent overuse of antibiotics in the management of watery diarrhoea in children in Abakaliki, Nigeria. BMC Infect Dis [Internet]. 2019 Dec 21;19(1):275. Available from: https://bmcinfectdis.biomedcentral.com/articles/10.1186/s12879-019-3899-1

27. Castellano VE, Giglio ND, Pacchiotti AC, Gentile Á. Outpatient management of childhood acute diarrhea: survey among pediatricians from a children's hospital in the Autonomous City of Buenos Aires. Arch Argent Pediatr [Internet]. 2022 Feb 1;120(1):46–53. Available from: https://www.sap.org.ar/docs/publicaciones/archivosarg/2022/v120n1a07e.pdf

28. Rheingans RD, Constenla D, Antil L, Innis BL, Breuer T. Economic and health burden of rotavirus gastroenteritis for the 2003 birth cohort in eight Latin American and Caribbean countries. Rev Panam Salud Publica/Pan Am J Public Heal. 2007;21(4):192–204.

29. Baral R, Nonvignon J, Debellut F, Agyemang SA, Clark A, Pecenka C. Cost of illness for childhood diarrhea in low- And middle-income countries: A systematic review of evidence

and modelled estimates. BMC Public Health. 2020;20(1):1–13.

30. Alvis-Guzman N, Orozco-Africano J, Paternina-Caicedo A, Coronell-Rodríguez W, Alvis-Estrada L, Jervis-Jálabe D, et al. Treatment costs of diarrheal disease and all-cause pneumonia among children under-5 years of age in Colombia. Vaccine [Internet]. 2013 Jul;31:C58–62. Available from: https://linkinghub.elsevier.com/retrieve/pii/S0264410X13006130

31. Burke RM, Smith ER, Dahl RM, Rebolledo PA, del Carmen Calderón M, Cañipa B, et al. The economic burden of pediatric gastroenteritis to Bolivian families: a cross-sectional study of correlates of catastrophic cost and overall cost burden. BMC Public Health [Internet]. 2014 Dec 24;14(1):642. Available from: http://bmcpublichealth.biomedcentral.com/articles/10.1186/1471-2458-14-642

32. Burke RM, Rebolledo PA, Embrey SR, Wagner LD, Cowden CL, Kelly FM, et al. The burden of pediatric diarrhea: a cross-sectional study of incurred costs and perceptions of cost among Bolivian families. BMC Public Health [Internet]. 2013 Dec 2;13(1):708. Available from: https://bmcpublichealth.biomedcentral.com/articles/10.1186/1471-2458-13-708

33. Jo C. Cost-of-illness studies: concepts, scopes, and methods. Clin Mol Hepatol [Internet]. 2014;20(4):327. Available from: http://e-cmh.org/journal/view.php?doi=10.3350/cmh.2014.20.4.327

34. Tarricone R. Cost-of-illness analysis. Health Policy (New York) [Internet]. 2006 Jun;77(1):51–63. Available from: https://linkinghub.elsevier.com/retrieve/pii/S0168851005001867

35. Sánchez Choez X, Loaiza Martínez M, Vaca Tatamuez V, López Peña M, Manzano Pasquel A, Jimbo Sotomayor R. Medical Cost of Upper Respiratory Tract Infections in

Children in Ambulatory Care. Value Heal Reg Issues [Internet]. 2021;26:1–9. Available from: http://dx.doi.org/10.1016/j.vhri.2020.10.001

36. Lambrechts T, Bryce J, Orinda V. Integrated management of childhood illness: a summary of first experiences. Bull World Health Organ [Internet]. 1999;77(7):582–94. Available from: http://www.ncbi.nlm.nih.gov/pubmed/10444882

37. Oliphant NP, Manda S, Daniels K, Odendaal WA, Besada D, Kinney M, et al. Integrated community case management of childhood illness in low- and middle-income countries. Cochrane Database Syst Rev [Internet]. 2021 Feb 10;2021(2). Available from: http://doi.wiley.com/10.1002/14651858.CD012882.pub2

38. Gera T, Shah D, Garner P, Richardson M, Sachdev HS. Integrated management of childhood illness (IMCI) strategy for children under five. Cochrane Database Syst Rev [Internet]. 2016 Jun 22; Available from: https://doi.wiley.com/10.1002/14651858.CD010123.pub2

39. Sánchez Choez X, Loaiza Martínez M, Vaca Tatamuez V, López Peña M, Manzano Pasquel A, Jimbo Sotomayor R. Medical Cost of Upper Respiratory Tract Infections in Children in Ambulatory Care. Value Heal Reg Issues [Internet]. 2021 Dec;26:1–9. Available from: https://doi.org/10.1016/j.vhri.2020.10.001

Supporting information

S1 File. Source and value of unit costs.

Click here to access/download
Supporting Information
S1 File Source and value of unit costs (1).pdf
