# Supplementary material for: Medical cost of acute diarrhea in children in ambulatory care
Source: PLoS One. 2022 Dec 16;17(12):e0279239. doi: 10.1371/journal.pone.0279239 (PMC9757569; doi:10.1371/journal.pone.0279239)
Supplement: Attachment — Submitted filename: PONE-D-22-19266_R1.pdf [file pone.0279239.s005.pdf]

PLOS ONE

Medical cost of acute diarrhea in children in ambulatory care

--Manuscript Draft--

**Manuscript Number:**	PONE-D-22-19266R1
**Article Type:**	Research Article
**Full Title:**	Medical cost of acute diarrhea in children in ambulatory care
**Short Title:**	Cost of Acute Diarrhea in Children
**Corresponding Author:**	Ruth Jimbo, PhD<br>Pontificia Universidad Catolica del Ecuador<br>Quito, Quito ECUADOR
**Keywords:**	Medical Cost, Diarrhea, Children, Ambulatory Care
**Abstract:**	Objective: The aim of this study was to estimate the direct medical cost per episode and the annual cost for acute diarrhea (AD) in children under five years of age in Ambulatory Care Centers of the Ministry of Public Health (MOPH) of Ecuador.<br>Methods: A cost of illness study with a provider perspective was carried out through a micro-costing of health resources and valuated in international dollars. Medical consultations and laboratory tests were valued using the tariff framework of services for the National Health System and for the prescribed medications, a reported cost registry of pharmacy purchases made in the year of study was used.<br>Results: A total of 332 electronic health records of children under five years of age were included in the analysis. Laboratory tests were performed on 37.95% (126/332), medications were prescribed to 93.67% (311/332) of the children, and antimicrobials were prescribed to 37.35% (124/332) of the children, representing an antibiotic prescription rate of 26.51% (88/332) and an antiparasitic prescription rate of 10.84% (36/332). The mean cost of the MOPH per child per episode of AD was US$45.24 (2019 dollars) (95% CI:43.71 to 46.76).<br>Conclusion: The total estimated cost of AD in children under five years of age for the MOPH in 2019 was about US$6,645,167.88 million (2019 dollars) (95% CI: 6,420,430.77 to 6,868,436.12). A high proportion of the direct medical cost of AD in children under five years of age in outpatient settings is due to unnecessary laboratory tests.
**Order of Authors:**	Xavier Sanchez Choez, PhD.
	Geraldine Leal Rojas, MD.
	Ángel Padilla Hernandez, MD.
	Ruth Jimbo-Sotomayor, PhD.
**Response to Reviewers:**	Academic Editor<br>“1. Please ensure that your manuscript meets PLOS ONE's style requirements, including those for file naming. The PLOS ONE style templates can be found at https://journals.plos.org/plosone/s/file?id=wjVg/PLOSOne_formatting_sample_main_body.pdf and https://journals.plos.org/plosone/s/file?id=ba62/PLOSOne_formatting_sample_title_authors_affiliations.pdf”<br>Corrections were made to meet PLOS ONE’s style requirements.<br>“2. Please update your submission to use the PLOS LaTeX template. The template and more information on our requirements for LaTeX submissions can be found at http://journals.plos.org/plosone/s/latex.”<br>PLOS template was used in this submission.<br>“3. Please include your full ethics statement in the ‘Methods’ section of your manuscript file. In your statement, please include the full name of the IRB or ethics committee who approved or waived your study, as well as whether or not you obtained informed written or verbal consent. If consent was waived for your study, please include this information in your statement as well.”<br>Full ethics statement was included in methods section. Lines 78-81<br>Reviewer #1:<br>“1. The whole manuscript should be revised for improved English and grammatical

Powered by Editorial Manager® and ProduXion Manager® from Aries Systems Corporation

mistakes.”
Suggestion accepted. The manuscript has been proofread by a native speaker.
“2. Line 38, %% should be corrected to %, similarly E.coli should be written as E.Coli. All bacterial pathogens name should be written in italic form.”
Suggestion accepted. Correction was made. Lines 52-53
“3. Line 79, ‘The district is distributed in 21 ambulatory medical care centres, These health centres are distributed in rural and urban areas of the city and offer health services to 85% of the population’. According to WHO criteria, 30 prescription per facility should be analysed. So the sample size should be at least 630.”
Observational burden of illness studies are widely used to characterize treatment patterns, resource utilization and costs associated with a disease. For a burden of illness study the aim of sample size calculation is to ensure sufficient precision in descriptive outcomes, e.g. characterized by the width of 95% confidence intervals (CIs). We followed sampling guidelines in cost-of-illness studies designed for practical application in real-world studies (1,2). The sampling approach used in our study complies with the methodological recommendations according to the guidelines for conducting health economics studies.

1.Johnston KM, Lakzadeh P, Donato BMK, Szabo SM. Methods of sample size calculation in descriptive retrospective burden of illness studies. BMC Med Res Methodol [Internet]. 2019;19(1):9. Available from: http://www.ncbi.nlm.nih.gov/pubmed/30626343
2.1. Jo C. Cost-of-illness studies: concepts, scopes, and methods. Clin Mol Hepatol [Internet]. 2014;20(4):327. Available from: http://e-cmh.org/journal/view.php?doi=10.3350/cmh.2014.20.4.327

“4. Line 123, Estimation of annual cost is not clear. How total number of AD episodes per year of the same patient was calculated?”
The annual cost was estimated as cost per episode per child in 2019 × total number of episodes in 2019. As this is a cost-of-illness study, all costs are imputed to each child to estimate an average cost. If the same child has had several episodes of the disease, this is already considered in the estimate. The methodology (1,2,3) for estimating disease costs provides an estimate of the burden resulting from the prevalence of the disease over a given period, most often a year. This is called the prevalence approach.

1.Hodgson TA, Meiners MR. Cost-of-Illness Methodology: A Guide to Current Practices and Procedures. Milbank Mem Fund Q Health Soc. 1982;60(3):429. Available from: https://www.jstor.org/stable/3349801?origin=crossref
2.Drummond MF: Methods for the economic evaluation of health care programmes. 3rd edition. Oxford: Oxford University Press; 2005.
3.Jo C. Cost-of-illness studies: concepts, scopes, and methods. Clin Mol Hepatol [Internet]. 2014;20(4):327. Available from: http://e-cmh.org/journal/view.php?doi=10.3350/cmh.2014.20.4.327

“5. Name of health facilities should be mentioned in a tabular form, mentioning the number of samples taken per facility.”
Suggestion accepted. Data is displayed in a Supplementary Table S1.
“6. I could not find the standard treatment of AD given by WHO. Please correlate your study with STGs of AD with WHO guidelines. You can add another variable of Adherence to STGs in your study. For your ease please read and cite the following article.
https://dx.doi.org/10.21608/bfsa.2020.93568”
The standard treatment of acute diarrhea is cited. A cost-of-illness study, such as this one, focuses on measuring the amount of resources used for a given disease and estimating the monetary expenditure. Therefore, a variable such as "adherence to standard treatment regimen" is not necessary (it is not a resource used). The implications derived from this variable go beyond our primary objective.
“7. et al should be italic.”
Suggestion accepted.
“8. Conclusion should be rewritten. As lab test is necessary so I don’t think that treatment cost is increased due to unnecessary testing. So revise conclusion according to prescribed medicines and its cost based on local or multinational brands.”
We politely disagree with the reviewer. According to the guidelines for treating acute diarrhea in children, laboratory testing is not necessary in the outpatient setting, as it will not modify the treatment. Therefore, the use of this resource reflects an unnecessary cost.
“9. No statistical test was applied on the variables.”
A cost-of-illness study, such as this one, focuses on measuring the amount of

Powered by Editorial Manager® and ProduXion Manager® from Aries Systems Corporation

	resources used for a given disease and estimating the monetary expenditure.<br>Advanced statistical tests are not necessary.<br>“Other comments:<br>The article focuses on treatment cost which is divided into two types, 1. Medication cost and 2. Lab test cost. On the other hand the article describes number of units dispensed from the facilities. So these two parameters should be clearly separated by headings and discussed accordingly in separate results heading.”<br>Suggestion accepted. Results were better discussed following the recommendation but meeting PLOS ONE’s style requirements<br><br>Reviewer #2:<br>“Authors should be more clear on the methodology and presentation of results in approach that can be understood by a layman. The comments provided in the attached pdf documents should be addressed clearly and correctly.”<br>Suggestion accepted. This manuscript version has been corrected and rewritten to facilitate comprehension.<br>All comments provided were properly addressed.<br>1.A definition of international dollar has been added. Lines 130-131.<br>2.Table 1 now includes information on comorbidities found in the sample. Line 152<br>3.Table 3 was reworked to improve comprehension. Line 176<br>4.US$ is used in the manuscript as the standard unit.<br><br>“This part is not clear and confusing. The prevalence rate is not the same as the sample size. I disagree that you can extrapolate the data on national prevalence to estimate annual cost. The annual cost should stay within your sample size. This is because health cost will vary by location. So, you can not assume the cost in your location is the same as elsewhere.”<br>Cost-of-illness studies can be described as prevalence-based or incidence-based approaches, depending on how the epidemiological data are used. Prevalence-based studies can use this procedure to extrapolate cost and resource data over a period of time (1,2). This is called a prevalence approach. We partially agree with the reviewer, as this type of estimation may have some limitations; however, this is discussed in lines 260-655. For this reason, it is advisable to perform a sensitivity analysis (lines 266-276).<br>1.Hodgson TA, Meiners MR. Cost-of-Illness Methodology: A Guide to Current Practices and Procedures. Milbank Mem Fund Q Health Soc. 1982;60(3):429. Available from: https://www.jstor.org/stable/3349801?origin=crossref<br>2.Jo C. Cost-of-illness studies: concepts, scopes, and methods. Clin Mol Hepatol [Internet]. 2014;20(4):327. Available from: http://e-cmh.org/journal/view.php?doi=10.3350/cmh.2014.20.4.327<br>“The term sensitivity analysis is not appropriate here. The information provided here can be included in the discussion but not discussed as sensitivity analysis”<br><br>Suggestion accepted. We discussed this analysis in the proper section. Lines 265-274.<br><br>“add " to the best of our knowledge"”<br>“Remove the word reliable as there is no way your audience can confirm that adjective. It is ok to write that you provided data on resources used but you don't need to stress the reliability. The reliability of resources may be difficult to measure in this context.”<br><br>Suggestions accepted. Lines 231-233<br>“If you say numerous studies, you should cite references backing that statement.”<br><br>Suggestion accepted. Lines 246-247<br>“Rather say the result may not be generalized since it is a limitation and not that it can be generalized.”<br>Suggestion accepted. We have rephrased the sentence to improve comprehension. Lines 291-293.
**Additional Information:**	
**Question**	**Response**
**Financial Disclosure**	The authors received no specific funding for this work.

Powered by Editorial Manager® and ProduXion Manager® from Aries Systems Corporation

Enter a financial disclosure statement that describes the sources of funding for the work included in this submission. Review the submission guidelines for detailed requirements. View published research articles from *PLOS ONE* for specific examples.<br><br>This statement is required for submission and **will appear in the published article** if the submission is accepted. Please make sure it is accurate.<br><br>**Unfunded studies**<br>Enter: *The author(s) received no specific funding for this work.*<br><br>**Funded studies**<br>Enter a statement with the following details:<br>• Initials of the authors who received each award<br>• Grant numbers awarded to each author<br>• The full name of each funder<br>• URL of each funder website<br>• Did the sponsors or funders play any role in the study design, data collection and analysis, decision to publish, or preparation of the manuscript?<br>• **NO** - Include this sentence at the end of your statement: *The funders had no role in study design, data collection and analysis, decision to publish, or preparation of the manuscript.*<br>• **YES** - Specify the role(s) played.<br><br>* typeset	
**Competing Interests**<br><br>Use the instructions below to enter a competing interest statement for this submission. On behalf of all authors, disclose any competing interests that could be perceived to bias this work—acknowledging all financial support and any other relevant financial or non-financial competing interests.<br><br>This statement is required for submission and **will appear in the published article** if the submission is accepted. Please make	The authors have declared that no competing interests exist.

sure it is accurate and that any funding sources listed in your Funding Information later in the submission form are also declared in your Financial Disclosure statement.<br><br>View published research articles from *PLOS ONE* for specific examples.<br><br>**NO authors have competing interests**<br><br>Enter: *The authors have declared that no competing interests exist.*<br><br>**Authors with competing interests**<br><br>Enter competing interest details beginning with this statement:<br><br>*I have read the journal's policy and the authors of this manuscript have the following competing interests: [insert competing interests here]*<br><br>* typeset	
**Ethics Statement**<br><br>Enter an ethics statement for this submission. This statement is required if the study involved:<br><br>• Human participants<br>• Human specimens or tissue<br>• Vertebrate animals or cephalopods<br>• Vertebrate embryos or tissues<br>• Field research<br><br>Write "N/A" if the submission does not require an ethics statement.<br><br>General guidance is provided below. Consult the submission guidelines for detailed instructions. **Make sure that all information entered here is included in the Methods section of the manuscript.**	Ethics approvals for the protocol and the study were granted by the Subcommittee for Research Ethics on Human Beings – Pontificia Universidad Católica del Ecuador with authorization code SB-CEISH-POS-749. The Ethics Committee established that there was no need for informed consent for this study.

Powered by Editorial Manager® and ProduXion Manager® from Aries Systems Corporation

**Format for specific study types**<br><br>**Human Subject Research (involving human participants and/or tissue)**<br>• Give the name of the institutional review board or ethics committee that approved the study<br>• Include the approval number and/or a statement indicating approval of this research<br>• Indicate the form of consent obtained (written/oral) or the reason that consent was not obtained (e.g. the data were analyzed anonymously)<br><br>**Animal Research (involving vertebrate animals, embryos or tissues)**<br>• Provide the name of the Institutional Animal Care and Use Committee (IACUC) or other relevant ethics board that reviewed the study protocol, and indicate whether they approved this research or granted a formal waiver of ethical approval<br>• Include an approval number if one was obtained<br>• If the study involved *non-human primates*, add *additional details* about animal welfare and steps taken to ameliorate suffering<br>• If anesthesia, euthanasia, or any kind of animal sacrifice is part of the study, include briefly which substances and/or methods were applied<br><br>**Field Research**<br><br>Include the following details if this study involves the collection of plant, animal, or other materials from a natural setting:<br>• Field permit number<br>• Name of the institution or relevant body that granted permission	
**Data Availability**<br><br>Authors are required to make all data underlying the findings described fully available, without restriction, and from the time of publication. PLOS allows rare exceptions to address legal and ethical concerns. See the PLOS Data Policy and FAQ for detailed information.	Yes - all data are fully available without restriction

A Data Availability Statement describing where the data can be found is required at submission. Your answers to this question constitute the Data Availability Statement and **will be published in the article**, if accepted.<br><br>**Important:** Stating 'data available on request from the author' is not sufficient. If your data are only available upon request, select 'No' for the first question and explain your exceptional situation in the text box.<br><br>Do the authors confirm that all data underlying the findings described in their manuscript are fully available without restriction?	
**Describe where the data may be found in full sentences. If you are copying our sample text, replace any instances of XXX with the appropriate details.**<br><br>• If the data are **held or will be held in a public repository**, include URLs, accession numbers or DOIs. If this information will only be available after acceptance, indicate this by ticking the box below. For example: *All XXX files are available from the XXX database (accession number(s) XXX, XXX.).*<br>• If the data are all contained **within the manuscript and/or Supporting Information files**, enter the following: *All relevant data are within the manuscript and its Supporting Information files.*<br>• If neither of these applies but you are able to provide **details of access elsewhere**, with or without limitations, please do so. For example:<br><br>*Data cannot be shared publicly because of [XXX]. Data are available from the XXX Institutional Data Access / Ethics Committee (contact via XXX) for researchers who meet the criteria for access to confidential data.*<br><br>*The data underlying the results presented in the study are available from (include the name of the third party*	All relevant data are within the manuscript and its Supporting Information files.

and contact information or URL).<br>• This text is appropriate if the data are owned by a third party and authors do not have permission to share the data.<br><br>* typeset	
Additional data availability information:	Tick here if the URLs/accession numbers/DOIs will be available only after acceptance of the manuscript for publication so that we can ensure their inclusion before publication.

Powered by Editorial Manager® and ProduXion Manager® from Aries Systems Corporation

Manuscript
Click here to access/download;Manuscript;Revised Manuscript.docx

Medical cost of acute diarrhea in children in ambulatory care

Xavier Sánchez[1,¶, #a], Gerardine Leal[2,&], Angel Padilla[2, &], Ruth Jimbo[1, ¶,*, #a]

[1]Centro de Investigación en Salud para América Latina (CISeAL), Facultad de Medicina, Pontificia Universidad Católica del Ecuador (PUCE), Quito, Ecuador

[2]Postgrado de Medicina Familiar y Comunitaria, Pontificia Universidad Católica del Ecuador (PUCE), Quito, Ecuador

[#a]Current address: Centro de Investigación para la Salud en América Latina (CISeAL), Pontificia Universidad Católica del Ecuador (PUCE), Quito, Ecuador

*Corresponding author:

E-mail: rejimbo@puce.edu.ec (RJ)

[¶]These authors contributed equally to this work.
[&]These authors also contributed equally to this work.

Abstract

Objective: The aim of this study was to estimate the direct medical cost per episode and the annual cost for acute diarrhea (AD) in children under five years of age in Ambulatory Care Centers of the Ministry of Public Health (MOPH) of Ecuador.

Methods: A cost of illness study with a provider perspective was carried out through a micro-costing of health resources and valuated in international dollars. Medical consultations and laboratory tests were valued using the tariff framework of services for the National Health System and for the prescribed medications, a reported cost registry of pharmacy purchases made in the year of study was used.

Results: A total of 332 electronic health records of children under five years of age were included in the analysis. Laboratory tests were performed on 37.95% (126/332), medications were prescribed to 93.67% (311/332) of the children, and antimicrobials were prescribed to 37.35% (124/332) of the children, representing an antibiotic prescription rate of 26.51% (88/332) and an antiparasitic prescription rate of 10.84% (36/332). The mean cost of the MOPH per child per episode of AD was US$45.24 (2019 dollars) (95% CI:43.71 to 46.76).

Conclusion: The total estimated cost of AD in children under five years of age for the MOPH in 2019 was about US$6,645,167.88 million (2019 dollars) (95% CI: 6,420,430.77 to 6,868,436.12). A high proportion of the direct medical cost of AD in children under five years of age in outpatient settings is due to unnecessary laboratory tests.

Introduction

Diarrheal disease is one of the leading causes of death in children in developing countries [1]. Acute diarrhea (AD) is defined as the passage of three or more loose or liquid stools per day [2]. It primarily occurs in children during the first five years after birth, and particularly in the second half-year and in little children [3]. It is estimated that 2.5 billion cases of diarrheal disease occur each year in children under five years of age, and an average of more than 1,400 children die each day [4]. Evidence shows that AD is common in places with poor access to health care, safe water and sanitation, which is often observed in low and middle-income countries [5].

The most common cause of AD is gastrointestinal infections caused by viruses, bacteria and occasionally parasite. Rotavirus is responsible for 60-70% of all diarrheal diseases; however, *E. coli*, *Campylobacter*, *Yersinia* and *Salmonella spp*. are common bacterial causes of AD [6]. The diagnosis of AD is mainly based on the medical interview, which provides accurate information about the duration of the diarrhea and the characteristics of the accompanying symptoms, such as abdominal pain, vomiting, and fever. Healthy children without any comorbidity do not require specific diagnosis tests in cases of AD since the results of laboratory and microbiological tests do not change the treatment of the episode; so, testing should be kept to a minimum and reserved for patients with severe illness [7].

Most episodes of AD are self-limiting, and prevention of dehydration is the main goal of diarrhea treatment. Medications such as antibiotics, antiparasitic drugs and antidiarrheal agents are not necessary and can be harmful for infants or children with diarrhea [8,9]. On occasion, antibiotics may be used in cases of bacterial infection when a specific cause of the diarrhea has been found or is strongly suspected, particularly after recent travel [10]. However, evidence shows that the most

commonly reported inappropriate measures used in AD treatment are the unwarranted prescription of antimicrobial, antidiarrheal and antimotility drugs, especially in low- and middle-income countries [11].

Unnecessary use of resources in the treatment of AD leads to inefficient use of scarce healthcare resources. It is estimated that a significant proportion of medical tests and drug prescriptions is unnecessary around the world; this is of particular concern because of the potential financial effect of excessive resource utilization on healthcare systems [12]. To date, no research has attempted to estimate this cost burden on Ecuador's healthcare system, even when AD is a leading cause of morbidity in children [13]. Demonstrating the importance of this problem to policy makers and clinicians would provide information to aid budgetary decision making and understanding of the need for cost containment interventions.

Materials and Methods

Ethics approvals for the protocol and the study were granted by the Subcommittee for Research Ethics on Human Beings – Pontificia Universidad Católica del Ecuador with authorization code SB-CEISH-POS-749. The Ethics Committee established that there was no need for informed consent for this study.

Objective

The aim of this study was to estimate the direct cost per episode and the annual cost of AD in the Ambulatory Care Centers of the Ministry of Public Health (MOPH) care system in 2019. Participants included children under five years of age, who had been diagnosed with AD.

Setting

Ecuador's health system is made up of two sectors, public and private. The MOPH is the governing entity of health in the country, offering health care services to the entire Ecuadorian population. Its financing comes from state contributions, and within the public health system it has a coverage of about 62%. The rest of the population is covered by a public subsystem such as social security institutions like the Ecuadorian Institute of Social Security (IESS), The Institute of Security of the Armed Forces (ISSFA) and the Institute of Social Security of the National Police (ISSPOL), or the private system in the case of segment of the population that has the means to pay [14,15].

District 17D03 of the city of Quito belongs to the public health network of Ecuador. This district has a total assigned population of 430,000 inhabitants and almost 36% is a pediatric population (0-18 years). The district is distributed in 21 ambulatory care centers; these health centers are distributed in rural and urban areas of the city and offer health services to 85% of the population [15,16].

Sample

A simple probabilistic type sampling was performed. To calculate the sample, the total number of cases registered by AD in the pediatric population under five reported by the MOPH in the district in 2019 was used, in Table S1. We considered an acute diarrhea diagnosis to be any diagnosis registered according to the ICD-10 classification, as the following: **A00:** Cholera, **A01:** Typhoid and paratyphoid fevers, **A02:** Other salmonella infections, **A03:** Shigellosis, **A04:** Other bacterial intestinal infections, **A05:** Other bacterial foodborne intoxication not elsewhere classified, **A06:**

Amoebiasis, **A07:** Other protozoal intestinal diseases, **A08:** Viral and other specified intestinal infections and **A09:** Other gastroenteritis and colitis of infectious and unspecified origin.

The following formula was applied to calculate the sample for a finite universe: $n = N*Z^2*p*q / d^2*(N-1) + Z^2*p*q$.

where N is the population size, Z is the confidence level (95%), p is the probability of success, or expected proportion (50%), q is the probability of failure (50%), and d is precision (5% of maximum admissible error in terms of proportion). The subsequently studied sample comprised 332 Electronic Health Records (EHR).

Identification of resource use and data collection

Perspective

A provider perspective was used, which included resources provided by the MOPH during the period between the initial consultation and resolution of the illness up to a maximum of five weeks. This included medical care, laboratory tests, and medication as primary resources.

Data source

The data source for the resources used and data collect in this study was the Electronic Health Records (EHR) of the patients from 21 health centers of the Ministry of Public Health in the District 17D03 in Ecuador during 2019 with AD as a primary diagnosis. This district has been using the EHR since 2010. Physicians enter information directly into the EHR on a computer during the outpatient appointment. Information was manually extracted from the EHR by two reviewers. The tests ordered and medication administered were verified against laboratory and pharmacy records.

Valuation of resources

All resources were valued in dollars at 2019 prices. To make the monetary values internationally comparable, they were converted to international dollars (a hypothetical currency with the same purchasing power of goods and services in all countries) using the purchasing power parity (PPP) conversion factor for Ecuador [17]. The cost of the resources was calculated based on information from official national cost sources. The National Health System service fee schedule and pharmacy drug purchase records were used [18]. Unit costs are available in Table S2

Estimating the annual cost for the MOPH

To estimate the annual cost of AD in 2019 for the MOPH in children under five years of age, the estimated cost per episode was combined with the total number of cases of AD treated by the MOPH at the first level of health for that population. The annual cost to the population was calculated as follows: *Annual cost 2019 = (cost per episode per child in 2019)* × *(total number of episodes in 2019).* The number of episodes in 2019 is the number of children under five years of age who were treated with AD in a primary health center of the MOPH in 2019, according to ICD-10 codes taken from the official of national statistics [13]. All analyses were carried out using SPSS v25 software.

Results

Participant characteristics

332 eligible EHR were randomly selected and proofed for suitability for inclusion in the cost-of-illness study. The general characteristics of our sample are described in Table 1.

Table 1. Characteristics of the sample.

Variable	n (%)
**Total electronic health records**	**332 (100)**
**Gender of patients**	
Female	141 (42.46)
Male	191 (57.53)
**Age of patients**	
Mean (SD)	1.85 (1.29)
**Comorbidity**	
No comorbidity	298 (89.76)
Chronic malnutrition	13 (3.92)
Anemia	11 (3.31)
Asthma	2 (0.60)
Obesity	2 (0.60)
Autism	1 (0.30)
Clift lip	1 (0.30)
Down’s syndrome	1 (0.30)
Epilepsy	1 (0.30)
Microcephaly	1 (0.30)
Prematurity	1 (0.30)
**Consultations by type of healthcare professional**	
Rural Doctor consultations	57 (17.16)
General Practitioner consultations	152 (45.78)
Family Medicine Physician consultations	39 (11.74)
Pediatrician consultations	84 (25.30)
**Total healthcare professionals**	**91 (100)**
**Gender of healthcare professionals**	
Female	59 (64.84)
Male	32 (35.16)
**Age of healthcare professionals**	
Mean (SD)	40.51 (13.41)
**Types of healthcare professionals**	
Rural Doctor	5 (5.49)
General Practitioner	57 (62.63)
Family Medicine Physician	26 (28.57)
Pediatrician	3 (3.29)

SD, standard deviation.

In the same of children, the male gender was more frequent with 57.53% (191/332) and the mean age was 1.29 years. Most of the registered children, 89.75% (198/332), had no comorbidities; chronic malnutrition and anemia were the most frequent comorbidities reported, with 3.92%

(13/332) and 3.31% (11/332) respectively. Of the total number of AD consultations, general practitioner consultations were more frequent with 45.78% (152/332) followed by pediatrician consultations with 25.30% (84/332).

A total of 91 healthcare professionals treated all the cases coded as AD, with females being the most frequent (64.84% vs. 35.16%) and the mean age of the professionals was 40.5. Two types of health care professionals were included: general physicians (Rural Doctor and General Practitioner) and specialists (Family Medicine Physician and Pediatrician), general physicians were the most frequent health professionals (68.12% vs. 31.88%).

The ICD-10 diagnostic code most frequently assigned by physicians was A09 (Other gastroenteritis and colitis of infectious and unspecified origin) in 98.19% (326/332) of the cases (Table 2).

Table 2. Consultations of acute diarrhea by type of healthcare professional.

**Health professional**	**ICD-10 diagnosis n (%)**				**Total**
	**A06.9**	**A07.1**	**A08.4**	**A09**	
Rural Doctor	0 (0)	0 (0)	0 (0)	57 (17.49)	57
General Practitioner	1 (25)	1 (100)	0 (0)	150 (46.01)	152
Family Medicine Physician	0 (0)	0 (0)	0 (0)	39 (11.96)	39
Pediatrician	3 (75)	0 (0)	1 (100)	80 (24.54)	84
**Total**	**4 (100)**	**1 (100)**	**1 (100)**	**326 (100)**	**332**

A06.9, Amoebiasis, unspecified; A07.1, Giardiasis [lambliasis]; A08.4, Viral intestinal infection, unspecified; A09, Other gastroenteritis and colitis of infectious and unspecified origin.

Resource use

The total resource use in the sample by item is shown in Table 3. A total of 1546 resources were used in 332 children. All children had one primary care contact, and the re-consultation rate was 9.94% (33/332); out of these, just one child needed two re-consultations.

Table 3. Resources used and mean cost per child

Direct sanitary resources	Number of patients	Units dispensed	Mean Cost per child (95%CI)[a]
**Consultations**			
First consultations	332	332	34.49 (34.40 – 34.55)
Re-consultations	33	34	22.88 (21.53 – 24.24)
**Laboratory tests**			
Blood count	38	38	5.10 (5.10 – 5.10)
Urinalysis	22	22	5.69 (5.69 – 5.69)
Gram stain & fresh drop	3	3	2.75 (2.75 – 2.75)
Coprological test	123	123	5.29 (5.29 – 5.29)
WBC, stool	80	80	2.55 (2.55 – 2.55)
Rotavirus	36	36	21.37 (21.37 – 21.37)
FOBT	18	18	3.14 (3.14 – 3.14)
**Prescriptions**			
Antibiotics	88	96	1.75 (1.27 – 2.22)
Antiparasitic	36	57	1.84 (1.65 – 2.02)
Anti-inflammatory	135	136	1.31 (1.25 – 1.37)
Oral rehydration salts	242	553	1.25 (1.18 – 1.33)
Zinc Sulfate	12	18	6.10 (4.59 – 7.61)
**Total cost**	**332**	**1546**	**45.24 (43.71 – 46.76)**

CI, Confident interval; WBC, Fecal leukocyte stain; FOBT, Fecal occult blood test.
[a]Cost presented in International Dollars 2019.

Laboratory tests were performed in 37.95% (126/332) children, representing 320 units of laboratory tests dispensed, of which 38.44% (123/320) were coprological tests, 25% (80/320) fecal leukocyte stain (WBC, stool), 11.25% (36/320) rotavirus tests, 11.88% (38/320) blood count, and 6.88% (22/320) urinalysis (Table 3); in some children, more than one test was ordered, the most frequent combination of tests were coprological test/fecal leukocyte stain in 63.49% (80/126) and coprological test/blood count at 35% (35/126).

Medications were prescribed to 93.67% (311/332) of children. Oral rehydration salts were the most prescribed medication, with 77.81% (242/311), followed by anti-inflammatory drugs with 43.41% (135/311). Antibiotics were prescribed to 88 children, representing an antibiotic prescription rate

of 26.51% (88/332) and antiparasitic were prescribed to 36 children, indicating a prescription rate of 10.84% (36/332). Zinc sulfate was prescribed to only 3.61% (12/332) (Table 3).

A total of 860 units of medication were dispensed, of these oral rehydration salts represented 60.30% (553/860) of the units used, anti-inflammatory drugs 17.79% (136/860), antibiotics 11.16% (96/860), antiparasitic 6.63% (57/860), and zinc sulfate 2.09% (18/860) (Table 3).

Medical contact accounts for 23.67% (366/1546) of the total resource use, prescriptions account for 55.63% (860/1546) and laboratory tests account for 20.70% (320/1546) (Table 4).

Table 4. Total resource use and overall cost of direct sanitary resources.

**Direct sanitary resources**	**Number of patients**	**Units dispensed**	**% Resource use**	**Overall Cost[a]**	**% Overall Cost[a]**
First consultations	332	332	21.47	1,1462.98	76.38
Re-consultations	33	34	2.20	755.29	5.03
Laboratory tests	126	320	20.70	2,007.74	13.38
Prescriptions	311	860	55.63	782.14	5.21
**Total cost**	**332**	**1546**	**100.00**	**15,008.15**	**100.00**

[a]Costs presented in International Dollars 2019.

Cost per episode and overall cost

The mean cost per child per resource used is displayed in Table 3. The mean cost to the MOPH per child per episode of AD was US$45.24 (2019 dollars) (95% CI:43.71, 46.76), the mean cost of laboratory tests was US$15.98 (95% CI: 14.20, 17.76) (2019 dollars), and the mean cost of drugs prescriptions was US$2.51 (95% CI: 2.27, 2.76) (2019 dollars).

The overall cost for the whole sample is presented in Table 4. The total cost of AD for the whole sample was US$15,008.15 (95% CI: 14,511.72, 15,524.32) (2019 dollars). The first consultation

accounted for 76.38% of the total cost of the sample and the subsequent consultations accounted for 5.03%. Laboratory tests accounted for 13.38% (US$2,007.74) (2019 dollars) and prescriptions accounted for 5.21% (US$782.14) (2019 dollars) of the overall cost.

The cost by type of laboratory test is shown in Table 5. Rotavirus tests accounted for 38.32% of the overall cost of laboratory tests, follow by coprological test with 32.41%, and white blood cells in stool test with 10.16%.

Table 5. Cost by type of laboratory test.

Type of laboratory test	Units	Total Cost[a]	%
Blood count	38	193.8	9.65
Urinalysis	22	125.18	6.23
Gram stain & fresh drop	3	8.25	0.41
Coprological test	123	650.67	32.41
WBC, stool test	80	204	10.16
Rotavirus	36	769.32	38.32
FOBT	18	56.52	2.82
**Total**	**320**	**2,007.74**	**100**

WBC, stool: Fecal leukocyte stain; FOBT, Fecal occult blood test.
[a]Costs presented in International Dollars 2019.

The cost by type of prescription is reflected in Table 6. Oral rehydration salts accounted for 38.82% of the overall cost of prescriptions, followed by any type of antimicrobial drug (antibiotic/antiparasitic) with 28.13%, analgesics/anti-inflammatory drugs with 22.73%, and zinc sulfate with 10.29%.

Table 6. Cost by type of medication

Type of medication	Units	Total Cost[a]	%
Amoxicillin	14	59.24	7.57
Azithromycin	9	20.94	2.68
Cephalexin	3	7.76	0.99
Clarithromycin	5	34.31	4.39

Trimethoprim/Sulfamethoxazole	65	31.86	4.07
Metronidazole	32	55.84	7.14
Albendazole	25	10.29	1.32
Ibuprofen	9	21.43	2.74
Paracetamol	127	156.37	19.99
Oral rehydration salts	553	303.63	38.82
Zinc Sulfate	18	80.47	10.29
**Total**	**860**	**782.14**	**100**

[a]Costs presented in International Dollars 2019.

Annual Cost to MOPH

We combined the cost per episode results with data on prevalence. The number of consultations for AD in children under five years of age in the ambulatory care centers of the MOPH, according to the National Direction of Statistics and Analysis of the Health Information (DNEAIS), in 2019 was 146,887 [19]. Thus, the annual cost to the MOPH was at least US$6,645,167.88 (2019 dollars) (95% CI: 6,420,430.77 to 6,868,436.12).

Discussion

To the best of our knowledge, this is the first study to measure AD management costs in children under five years of age in Ecuador. Our data provide statistics on resource use and costs from a payer perspective. The cost was estimated from medical records with diagnoses coded according to ICD-10, applying a micro-cost methodology by involving the direct identification and valuation of the resources consumed treating each patient, which improves the accuracy of cost estimation [20].

Medical appointments represent 23.67% of the resources used per AD episode. All children had at least an initial consultation and the rate of re-consultation was almost 10%, which is less than that reported in the literature [21]. Laboratory tests were ordered in almost 38% of the children, accounting for around the 20% of the resources used.

Medication prescription was the most frequently used resource in the treatment of AD cases. It accounted for 55.63% of all resource units administered. Oral rehydration salts, anti-inflammatory drugs and antibiotics were the most prescribed drugs, 72.89%, 40.66%, and 26.51% respectively; these results are similar to other studies [22–24]. Regardless of this, it is important to mention that the use of resources in medical care may vary according to their availability.

Numerous studies have reported the economic burden of childhood diarrhea in different settings; however, empirical data on the cost of diarrheal illness is sparse [25–29]. We found that the average cost of AD per episode in children under five years of age in Ecuador was US$45.24 (2019 dollars). Our results are similar to those of some countries reported by Rheingans *et al.* [30] for Latin American and Caribbean countries, where total direct medical cost in outpatient settings was US$18.45 in Argentina, US$15.03 in Brazil, 47.25 US$ in Chile, US$30.26 in Honduras, US$33.58 in Mexico, US$39.50 in Panamá and US$30.96 in Venezuela (monetary change to 2019). Nevertheless, the proportion of laboratory and medication costs were higher in Rheingans' study, between 50% and 80%, compared to our data, which was less than 20%. Alternatively, a systematic review of the cost of childhood diarrhea in 137 low- and middle-income countries (LMICs) estimated an average (weighted) US$52.16 per outpatient episode of AD [31], and the direct medical cost represented almost 80% of the total cost, US$41.21 (2020 dollars). These findings are more comparable with our results.

Considerable variation is found in the cost estimates reported in the literature, variation that can be attributed to methodological differences between studies, in particular variation in the perspective adopted by the studies and the categorization of the costs [32–34]. Additionally, the direct cost associated with treatment could vary according to the capabilities and resources available in

different health units, even within the same country, leading to some heterogeneity in the estimated costs of the studies.

The results from our study on the cost of illness may be conservative. As part of the sensitivity analysis, the effect of the proportion of laboratory tests was explored. Otherwise healthy children with AD in ambulatory settings do not require a specific diagnostic work-up, as the results of laboratory investigations are unlikely to change their management [10,35]. According to the Integrated Management of Childhood Illness (IMCI)[36], among other components, to improve the performance of health personnel caring for children under five, it is not necessary to request laboratory tests in outpatient care. However, we found a proportion of laboratory tests ordered of around 13%. If we consider that physicians comply with the recommendations for care according to IMCI, the estimated population cost would be US$ 800,000 lower. Other variables in the study do not greatly affect the results as we found them to be relatively low cost.

Cost-of-illness studies can estimate cost based on epidemiological data, through incidence-based or prevalence-based approaches [37]; the latter is useful for identifying points for cost containment, as this approach provides information on each cost component [38,39].

The total estimated cost of AD in children under five years of age for MOPH in 2019 was about US$6,645,167.88 (2019 dollars) (95% CI: 6,420,430.77 to 6,868,436.12). If laboratory tests had been minimized and antibiotic use cut by at least half, our annual cost estimate would have been reduced by $1 million. Therefore, an intervention that promotes rational use of these resources would be cost saving.

The Integrated Management of Childhood Illness (IMCI) strategy was designed in 1996 by the World Health Organization (WHO) and the United Nations Children's Fund (UNICEF) to improve

the quality of care for children in primary care services and was adopted by Ecuador [40]. According to this strategy, in patients under five years of age with AD, it is not necessary to request laboratory tests, and the use of antibiotics is justified in few circumstances; therefore, our findings show that there may be an overuse of these resources and lack of adherence to IMCI recommendations, a situation that has been reported in other studies [41,42]. However, the evaluation of the strategy and its implementation is beyond our research objectives.

A limitation of this study is that it focuses only on the population subsidized by the MOPH; although it covers most of the country's population[43], the results may not be generalizable to other public health subsystems. Another limitation is the perspective used in the study; we did not include patient time costs or other associated resource costs. This perspective is narrower and does not include patients' out-of-pocket costs as they are not borne by payers; therefore, the cost of illness may be underestimated. A strength of the study is that the AD cases were selected from the medical records of different health centers, according to ICD-10, which allowed us to obtain data according to their own diagnoses and thus determine the use of resources for such care. Moreover, the study shows relevant data for decision-making stakeholders to consider cost containment measures for the efficient use of medical resources.

Conclusion:

A high proportion of the direct medical cost of AD in children under five years of age in outpatient settings is due to the unnecessary use of laboratory tests. Factors that may influence the overuse of this resource in primary care need to be explored.

Acknowledgements:

None

References:

1. Hug L, Sharrow D, Danzhen Y, on behalf of the United Nations Inter-agency Group for Child Mortality Estimation (UN IGME). Levels & Trends in Child Mortality: Report 2019. UNICEF [Internet]. 2019;52. Available from: https://www.unicef.org/media/60561/file/UN-IGME-child-mortality-report-2019.pdf

2. Drancourt M. Acute Diarrhea. In: Infectious Diseases [Internet]. Elsevier; 2017. p. 335-340.e2. Available from: https://linkinghub.elsevier.com/retrieve/pii/B9780702062858000381

3. Radlovic N, Lekovic Z, Vuletic B, Radlovic V, Simic D. Acute diarrhea in children. Srp Arh Celok Lek [Internet]. 2015;143(11–12):755–62. Available from: http://www.doiserbia.nb.rs/Article.aspx?ID=0370-81791512755R

4. Pinzón-Rondón ÁM, Zárate-Ardila C, Hoyos-Martínez A, Ruiz-Sternberg ÁM, Vélez-van-Meerbeke A. Country characteristics and acute diarrhea in children from developing nations: a multilevel study. BMC Public Health [Internet]. 2015 Dec 21;15(1):811. Available from: http://bmcpublichealth.biomedcentral.com/articles/10.1186/s12889-015-2120-8

5. Troeger C, Forouzanfar M, Rao PC, Khalil I, Brown A, Reiner RC, et al. Estimates of global, regional, and national morbidity, mortality, and aetiologies of diarrhoeal diseases: a systematic analysis for the Global Burden of Disease Study 2015. Lancet Infect Dis [Internet]. 2017 Sep;17(9):909–48. Available from: https://linkinghub.elsevier.com/retrieve/pii/S1473309917302761

6. Cheng AC, McDonald JR, Thielman NM. Infectious Diarrhea in Developed and

Developing Countries. J Clin Gastroenterol [Internet]. 2005 Oct;39(9):757–73. Available from: http://journals.lww.com/00004836-200510000-00002

7. Aranda-Michel J, Giannella RA. Acute diarrhea: a practical review. Am J Med [Internet]. 1999 Jun;106(6):670–6. Available from: https://linkinghub.elsevier.com/retrieve/pii/S000293439900128X

8. Florez ID, Veroniki A-A, Al Khalifah R, Yepes-Nuñez JJ, Sierra JM, Vernooij RWM, et al. Comparative effectiveness and safety of interventions for acute diarrhea and gastroenteritis in children: A systematic review and network meta-analysis. van Wouwe JP, editor. PLoS One [Internet]. 2018 Dec 5;13(12):e0207701. Available from: https://dx.plos.org/10.1371/journal.pone.0207701

9. Lo Vecchio A, Dias JA, Berkley JA, Boey C, Cohen MB, Cruchet S, et al. Comparison of Recommendations in Clinical Practice Guidelines for Acute Gastroenteritis in Children. J Pediatr Gastroenterol Nutr [Internet]. 2016 Aug;63(2):226–35. Available from: https://journals.lww.com/00005176-201608000-00011

10. Guarino A, Lo Vecchio A, Dias JA, Berkley JA, Boey C, Bruzzese D, et al. Universal Recommendations for the Management of Acute Diarrhea in Nonmalnourished Children. J Pediatr Gastroenterol Nutr [Internet]. 2018 Nov;67(5):586–93. Available from: https://journals.lww.com/00005176-201811000-00011

11. Carter E, Bryce J, Perin J, Newby H. Harmful practices in the management of childhood diarrhea in low- and middle-income countries: a systematic review. BMC Public Health [Internet]. 2015 Dec 18;15(1):788. Available from: http://bmcpublichealth.biomedcentral.com/articles/10.1186/s12889-015-2127-1

12. Brownlee S, Chalkidou K, Doust J, Elshaug AG, Glasziou P, Heath I, et al. Evidence for overuse of medical services around the world. Lancet [Internet]. 2017

Jul;390(10090):156–68. Available from: https://linkinghub.elsevier.com/retrieve/pii/S0140673616325855

13. Ministerio de Salud Pública del Ecuador. Información Estadística y Geográfica de Salud [Internet]. 2017 [cited 2020 Jun 7]. Available from: https://www.salud.gob.ec/informacion-estadistica-de-produccion-de-salud/#

14. Lucio R, Villacrés N, Henríquez R. Sistema de salud de Ecuador. Salud Publica Mex [Internet]. 2011 [cited 2015 Jan 15];53(1):177–87. Available from: http://www.scielo.org.mx/scielo.php?pid=S0036-36342011000800013&script=sci_arttext

15. Instituto Nacional de Estadísticas y Censos (INEC-Ecuador). Encuesta de Condiciones de Vida. ECV [Internet]. 2015. Available from: https://www.ecuadorencifras.gob.ec/encuesta-de-condiciones-de-vida-ecv

16. Ministerio de Salud Pública del Ecuador. Manual del Modelo de Atencion Integral del Sistema Nacional de Salud Familiar Comunitario e Intercultural (MAIS-FCI) [Internet]. 2012 [cited 2019 Jun 28]. Available from: http://instituciones.msp.gob.ec/somossalud/images/documentos/guia/Manual_MAIS-MSP12.12.12.pdf

17. International Monetary Fund. World Economic Outlook Database. 2017; Available from: https://www.imf.org/external/pubs/ft/weo/2017/02/weodata/index.aspx

18. Ministerio de Salud Pública del Ecuador. Tarifario de Prestaciones para el Sistema Nacional de Salud [Internet]. 2014. Available from: https://aplicaciones.msp.gob.ec/salud/archivosdigitales/documentosDirecciones/dnn/archivos/TARIFARIO.pdf

19. Dirección Nacional de Estadística y Análisis de Información de Salud – Ministerio de Salud Pública. Registro Diario Automatizado de Consultas y Atenciones Ambulatorias

(RDACAA 1.6 y RDACAA 2.0) y Plataforma de registro en atenciones de salud (PRAS) [Internet]. 2019 [cited 2022 Apr 21]. Available from: https://www.salud.gob.ec/direccion-nacional-de-estadistica-y-analisis-de-informacion-de-salud/

20. Xu X, Grossetta Nardini HK, Ruger JP. Micro-costing studies in the health and medical literature: protocol for a systematic review. Syst Rev [Internet]. 2014 Dec 21;3(1):47. Available from: https://systematicreviewsjournal.biomedcentral.com/articles/10.1186/2046-4053-3-47

21. Rautenberg TA. Evaluating the cost utility of racecadotril for the treatment of acute watery diarrhea in children: the RAWD model. Clin Outcomes Res [Internet]. 2012 Apr;109. Available from: http://www.dovepress.com/evaluating-the-cost-utility-of-racecadotril-for-the-treatment-of-acute-peer-reviewed-article-CEOR

22. Okubo Y, Miyairi I, Michihata N, Morisaki N, Kinoshita N, Urayama KY, et al. Recent Prescription Patterns for Children With Acute Infectious Diarrhea. J Pediatr Gastroenterol Nutr [Internet]. 2019 Jan;68(1):13–6. Available from: https://journals.lww.com/00005176-201901000-00004

23. Efunshile AM, Ezeanosike O, Nwangwu CC, König B, Jokelainen P, Robertson LJ. Apparent overuse of antibiotics in the management of watery diarrhoea in children in Abakaliki, Nigeria. BMC Infect Dis [Internet]. 2019 Dec 21;19(1):275. Available from: https://bmcinfectdis.biomedcentral.com/articles/10.1186/s12879-019-3899-1

24. Castellano VE, Giglio ND, Pacchiotti AC, Gentile Á. Outpatient management of childhood acute diarrhea: survey among pediatricians from a children's hospital in the City of Buenos Aires. Arch Argent Pediatr [Internet]. 2022 Feb 1;120(1):46–53. Available from: https://www.sap.org.ar/docs/publicaciones/archivosarg/2022/v120n1a07e.pdf

25. Ehrenkranz P, Lanata CF, Penny ME, Salazar-Lindo E, Glass RI. Rotavirus diarrhea

disease burden in Peru: the need for a rotavirus vaccine and its potential cost savings. Rev Panam Salud Pública [Internet]. 2001 Oct;10(4):240–8. Available from: http://www.scielosp.org/scielo.php?script=sci_arttext&pid=S1020-49892001001000004&lng=en&nrm=iso&tlng=en

26. Rochanathimoke O, Riewpaiboon A, Tharmaphornpilas P, Jiamsiri S, Thavorncharoensap M, Postma MJ. Economic burden of rotavirus diarrhea in Thailand: Report from a pilot study on rotavirus vaccination. Vaccine [Internet]. 2019 Jan;37(4):587–94. Available from: https://linkinghub.elsevier.com/retrieve/pii/S0264410X18316736

27. Ngabo F, Mvundura M, Gazley L, Gatera M, Rugambwa C, Kayonga E, et al. The Economic Burden Attributable to a Child's Inpatient Admission for Diarrheal Disease in Rwanda. Griffiths UK, editor. PLoS One [Internet]. 2016 Feb 22;11(2):e0149805. Available from: https://dx.plos.org/10.1371/journal.pone.0149805

28. Hasan MZ, Mehdi GG, De Broucker G, Ahmed S, Ali MW, Martin Del Campo J, et al. The economic burden of diarrhea in children under 5 years in Bangladesh. Int J Infect Dis [Internet]. 2021 Jun;107:37–46. Available from: https://linkinghub.elsevier.com/retrieve/pii/S1201971221003490

29. Rheingans R, Anderson JD, Bagamian KH, Laytner LA, Pecenka CJ, Gilani SSA, et al. Effects of geographic and economic heterogeneity on the burden of rotavirus diarrhea and the impact and cost-effectiveness of vaccination in Pakistan. Vaccine [Internet]. 2018 Dec;36(51):7780–9. Available from: https://linkinghub.elsevier.com/retrieve/pii/S0264410X18301725

30. Rheingans RD, Constenla D, Antil L, Innis BL, Breuer T. Economic and health burden of rotavirus gastroenteritis for the 2003 birth cohort in eight Latin American and Caribbean countries. Rev Panam Salud Publica/Pan Am J Public Heal. 2007;21(4):192–204.

31. Baral R, Nonvignon J, Debellut F, Agyemang SA, Clark A, Pecenka C. Cost of illness for childhood diarrhea in low- And middle-income countries: A systematic review of evidence and modelled estimates. BMC Public Health. 2020;20(1):1–13.

32. Alvis-Guzman N, Orozco-Africano J, Paternina-Caicedo A, Coronell-Rodríguez W, Alvis-Estrada L, Jervis-Jálabe D, et al. Treatment costs of diarrheal disease and all-cause pneumonia among children under-5 years of age in Colombia. Vaccine [Internet]. 2013 Jul;31:C58–62. Available from: https://linkinghub.elsevier.com/retrieve/pii/S0264410X13006130

33. Burke RM, Smith ER, Dahl RM, Rebolledo PA, del Carmen Calderón M, Cañipa B, et al. The economic burden of pediatric gastroenteritis to Bolivian families: a cross-sectional study of correlates of catastrophic cost and overall cost burden. BMC Public Health [Internet]. 2014 Dec 24;14(1):642. Available from: http://bmcpublichealth.biomedcentral.com/articles/10.1186/1471-2458-14-642

34. Burke RM, Rebolledo PA, Embrey SR, Wagner LD, Cowden CL, Kelly FM, et al. The burden of pediatric diarrhea: a cross-sectional study of incurred costs and perceptions of cost among Bolivian families. BMC Public Health [Internet]. 2013 Dec 2;13(1):708. Available from: https://bmcpublichealth.biomedcentral.com/articles/10.1186/1471-2458-13-708

35. Florez ID, Niño-Serna LF, Beltrán-Arroyave CP. Acute Infectious Diarrhea and Gastroenteritis in Children. Curr Infect Dis Rep [Internet]. 2020 Feb 28;22(2):4. Available from: http://link.springer.com/10.1007/s11908-020-0713-6

36. Ministerio de Salud Pública del Ecudor - Dirección Nacional de Normatización. Atención Integrada a Enfermedades Prevalentes de la Infancia (AIEPI) Clínico. Cuadro de Procedimientos. Actualización 2017 [Internet]. Quito; 2017. 92 p. Available from:

http://instituciones.msp.gob.ec/images/Documentos/AIEPI_6feb_2019_web.pdf
37. Jo C. Cost-of-illness studies: concepts, scopes, and methods. Clin Mol Hepatol [Internet]. 2014;20(4):327. Available from: http://e-cmh.org/journal/view.php?doi=10.3350/cmh.2014.20.4.327
38. Tarricone R. Cost-of-illness analysis. Health Policy (New York) [Internet]. 2006 Jun;77(1):51–63. Available from: https://linkinghub.elsevier.com/retrieve/pii/S0168851005001867
39. Sánchez Choez X, Loaiza Martínez M, Vaca Tatamuez V, López Peña M, Manzano Pasquel A, Jimbo Sotomayor R. Medical Cost of Upper Respiratory Tract Infections in Children in Ambulatory Care. Value Heal Reg Issues [Internet]. 2021;26:1–9. Available from: http://dx.doi.org/10.1016/j.vhri.2020.10.001
40. Lambrechts T, Bryce J, Orinda V. Integrated management of childhood illness: a summary of first experiences. Bull World Health Organ [Internet]. 1999;77(7):582–94. Available from: http://www.ncbi.nlm.nih.gov/pubmed/10444882
41. Oliphant NP, Manda S, Daniels K, Odendaal WA, Besada D, Kinney M, et al. Integrated community case management of childhood illness in low- and middle-income countries. Cochrane Database Syst Rev [Internet]. 2021 Feb 10;2021(2). Available from: http://doi.wiley.com/10.1002/14651858.CD012882.pub2
42. Gera T, Shah D, Garner P, Richardson M, Sachdev HS. Integrated management of childhood illness (IMCI) strategy for children under five. Cochrane Database Syst Rev [Internet]. 2016 Jun 22; Available from: https://doi.wiley.com/10.1002/14651858.CD010123.pub2
43. Ministerio de Salud Pública del Ecuador. En 2018: se evidenció un Sistema Nacional de Salud fortalecido – Ministerio de Salud Pública [Internet]. 2019 [cited 2022 Jun 3].

Available from: https://www.salud.gob.ec/en-2018-se-evidencio-un-sistema-nacional-de-salud-fortalecido/

Supporting information

S1 Table. Cases registered as acute diarrhea in the pediatric population under five years of age

S2 Table. Source and value of unit costs

Supporting Information S1 Table

Click here to access/download
Supporting Information
S1_Table.docx

Supporting Information S2 Table

Click here to access/download
Supporting Information
S2_Table.docx

Manuscript with track changes

Medical cost of acute diarrhea in children in ambulatory care

Xavier Sánchez[1,¶, #a] , Gerardine Leal[2,&], Angel Padilla[2, &], Ruth Jimbo[1, ¶,*, #a]

[1]Centro de Investigación en Salud para América Latina (CISeAL), Facultad de Medicina, Pontificia Universidad Católica del Ecuador (PUCE), Quito, Ecuador

[2]Postgrado de Medicina Familiar y Comunitaria, Pontificia Universidad Católica del Ecuador (PUCE), Quito, Ecuador

[#a]Current address: Centro de Investigación para la Salud en América Latina (CISeAL), Pontificia Universidad Católica del Ecuador (PUCE), Quito, Ecuador

*Corresponding author:

E-mail: rejimbo@puce.edu.ec (RJ)

[¶]These authors contributed equally to this work.
[&]These authors also contributed equally to this work.

Abstract

Objective: The aim of this study was to estimate the direct medical cost per episode and the annual cost for acute diarrhea (AD) in children under five years of age in Ambulatory Care Centers of the Ministry of Public Health (MOPH) of Ecuador.

Methods: A cost of illness study with a provider perspective was carried out through a micro-costing of health resources and valuated in international dollars. Medical consultations and laboratory tests were valued using the tariff framework of services for the National Health System and for the prescribed medications, a reported cost registry of pharmacy purchases made in the year of study was used.

Results: A total of 332 electronic health records of children under five years of age were included in the analysis. Laboratory tests were performed on 37.95% (126/332), medications were prescribed to 93.67% (311/332) of the children, and antimicrobials were prescribed to 37.35% (124/332) of the children, representing an antibiotic prescription rate of 26.51% (88/332) and an antiparasitic prescription rate of 10.84% (36/332). The mean cost of the MOPH per child per episode of AD was US$45.24 (2019 dollars) (95% CI:43.71 to 46.76).

Conclusion: The total estimated cost of AD in children under five years of age for the MOPH in 2019 was about US$6,645,167.88 million (2019 dollars) (95% CI: 6,420,430.77 to 6,868,436.12). A high proportion of the direct medical cost of AD in children under five years of age in outpatient settings is due to unnecessary laboratory tests.

Introduction

Diarrheal disease is one of the leading causes of death in children in developing countries [1]. Acute diarrhea (AD) is defined as the passage of three or more loose or liquid stools per day [2]. It primarily occurs in children during the first five years after birth, and particularly in the second half-year and in little children [3]. It is estimated that 2.5 billion cases of diarrheal disease occur each year in children under five years of age, and an average of more than 1,400 children die each day [4]. Evidence shows that AD is common in places with poor access to health care, safe water and sanitation, which is often observed in low and middle-income countries [5].

The most common cause of AD is gastrointestinal infections caused by viruses, bacteria and occasionally parasite. Rotavirus is responsible for 60-70% of all diarrheal diseases; however, *E. coli, Campylobacter, Yersinia* and *Salmonella spp*. are common bacterial causes of AD [6]. The diagnosis of AD is mainly based on the medical interview, which provides accurate information about the duration of the diarrhea and the characteristics of the accompanying symptoms, such as abdominal pain, vomiting, and fever. Healthy children without any comorbidity do not require specific diagnosis tests in cases of AD since the results of laboratory and microbiological tests do not change the treatment of the episode; so, testing should be kept to a minimum and reserved for patients with severe illness [7].

Most episodes of AD are self-limiting, and prevention of dehydration is the main goal of diarrhea treatment. Medications such as antibiotics, antiparasitic drugs and antidiarrheal agents are not necessary and can be harmful for infants or children with diarrhea [8,9]. On occasion, antibiotics may be used in cases of bacterial infection when a specific cause of the diarrhea has been found or is strongly suspected, particularly after recent travel [10]. However, evidence shows that the most

commonly reported inappropriate measures used in AD treatment are the unwarranted prescription of antimicrobial, antidiarrheal and antimotility drugs, especially in low- and middle-income countries [11].

Unnecessary use of resources in the treatment of AD leads to inefficient use of scarce healthcare resources. It is estimated that a significant proportion of medical tests and drug prescriptions is unnecessary around the world; this is of particular concern because of the potential financial effect of excessive resource utilization on healthcare systems [12]. To date, no research has attempted to estimate this cost burden on Ecuador's healthcare system, even when AD is a leading cause of morbidity in children [13]. Demonstrating the importance of this problem to policy makers and clinicians would provide information to aid budgetary decision making and understanding of the need for cost containment interventions.

Materials and Methods

Ethics approvals for the protocol and the study were granted by the Subcommittee for Research Ethics on Human Beings – Pontificia Universidad Católica del Ecuador with authorization code SB-CEISH-POS-749. The Ethics Committee established that there was no need for informed consent for this study.

Objective

The aim of this study was to estimate the direct cost per episode and the annual cost of AD in the Ambulatory Care Centers of the Ministry of Public Health (MOPH) care system in 2019. Participants included children under five years of age, who had been diagnosed with AD.

Setting

Ecuador's health system is made up of two sectors, public and private. The MOPH is the governing entity of health in the country, offering health care services to the entire Ecuadorian population. Its financing comes from state contributions, and within the public health system it has a coverage of about 62%. The rest of the population is covered by a public subsystem such as social security institutions like the Ecuadorian Institute of Social Security (IESS), The Institute of Security of the Armed Forces (ISSFA) and the Institute of Social Security of the National Police (ISSPOL), or the private system in the case of segment of the population that has the means to pay [14,15].

District 17D03 of the city of Quito belongs to the public health network of Ecuador. This district has a total assigned population of 430,000 inhabitants and almost 36% is a pediatric population (0-18 years). The district is distributed in 21 ambulatory care centers; these health centers are distributed in rural and urban areas of the city and offer health services to 85% of the population [15,16].

Sample

A simple probabilistic type sampling was performed. To calculate the sample, the total number of cases registered by AD in the pediatric population under five reported by the MOPH in the district in 2019 was used, in Table S1. We considered an acute diarrhea diagnosis to be any diagnosis registered according to the ICD-10 classification, as the following: **A00:** Cholera, **A01:** Typhoid and paratyphoid fevers, **A02:** Other salmonella infections, **A03:** Shigellosis, **A04:** Other bacterial intestinal infections, **A05:** Other bacterial foodborne intoxication not elsewhere classified, **A06:**

Amoebiasis, **A07:** Other protozoal intestinal diseases, **A08:** Viral and other specified intestinal infections and **A09:** Other gastroenteritis and colitis of infectious and unspecified origin.

The following formula was applied to calculate the sample for a finite universe: $n = N*Z^2*p*q / d^2*(N-1) + Z^2*p*q$.

where N is the population size, Z is the confidence level (95%), p is the probability of success, or expected proportion (50%), q is the probability of failure (50%), and d is precision (5% of maximum admissible error in terms of proportion). The subsequently studied sample comprised 332 Electronic Health Records (EHR).

Identification of resource use and data collection

Perspective

A provider perspective was used, which included resources provided by the MOPH during the period between the initial consultation and resolution of the illness up to a maximum of five weeks. This included medical care, laboratory tests, and medication as primary resources.

Data source

The data source for the resources used and data collect in this study was the Electronic Health Records (EHR) of the patients from 21 health centers of the Ministry of Public Health in the District 17D03 in Ecuador during 2019 with AD as a primary diagnosis. This district has been using the EHR since 2010. Physicians enter information directly into the EHR on a computer during the outpatient appointment. Information was manually extracted from the EHR by two reviewers. The tests ordered and medication administered were verified against laboratory and pharmacy records.

Valuation of resources

All resources were valued in dollars at 2019 prices. To make the monetary values internationally comparable, they were converted to international dollars (a hypothetical currency with the same purchasing power of goods and services in all countries) using the purchasing power parity (PPP) conversion factor for Ecuador [17]. The cost of the resources was calculated based on information from official national cost sources. The National Health System service fee schedule and pharmacy drug purchase records were used [18]. Unit costs are available in Table S2

Estimating the annual cost for the MOPH

To estimate the annual cost of AD in 2019 for the MOPH in children under five years of age, the estimated cost per episode was combined with the total number of cases of AD treated by the MOPH at the first level of health for that population. The annual cost to the population was calculated as follows: *Annual cost 2019 = (cost per episode per child in 2019)* × *(total number of episodes in 2019).* The number of episodes in 2019 is the number of children under five years of age who were treated with AD in a primary health center of the MOPH in 2019, according to ICD-10 codes taken from the official of national statistics [13]. All analyses were carried out using SPSS v25 software.

Results

Participant characteristics

332 eligible EHR were randomly selected and proofed for suitability for inclusion in the cost-of-illness study. The general characteristics of our sample are described in Table 1.

Table 1. Characteristics of the sample.

Variable	n (%)
**Total electronic health records**	**332 (100)**
**Gender of patients**	
Female	141 (42.46)
Male	191 (57.53)
**Age of patients**	
Mean (SD)	1.85 (1.29)
**Comorbidity**	
No comorbidity	298 (89.76)
Chronic malnutrition	13 (3.92)
Anemia	11 (3.31)
Asthma	2 (0.60)
Obesity	2 (0.60)
Autism	1 (0.30)
Clift lip	1 (0.30)
Down's syndrome	1 (0.30)
Epilepsy	1 (0.30)
Microcephaly	1 (0.30)
Prematurity	1 (0.30)
**Consultations by type of healthcare professional**	
Rural Doctor consultations	57 (17.16)
General Practitioner consultations	152 (45.78)
Family Medicine Physician consultations	39 (11.74)
Pediatrician consultations	84 (25.30)
**Total healthcare professionals**	**91 (100)**
**Gender of healthcare professionals**	
Female	59 (64.84)
Male	32 (35.16)
**Age of healthcare professionals**	
Mean (SD)	40.51 (13.41)
**Types of healthcare professionals**	
Rural Doctor	5 (5.49)
General Practitioner	57 (62.63)
Family Medicine Physician	26 (28.57)
Pediatrician	3 (3.29)

SD, standard deviation.

In the same of children, the male gender was more frequent with 57.53% (191/332) and the mean age was 1.29 years. Most of the registered children, 89.75% (198/332), had no comorbidities;

chronic malnutrition and anemia were the most frequent comorbidities reported, with 3.92% (13/332) and 3.31% (11/332) respectively. Of the total number of AD consultations, general practitioner consultations were more frequent with 45.78% (152/332) followed by pediatrician consultations with 25.30% (84/332).

A total of 91 healthcare professionals treated all the cases coded as AD, with females being the most frequent (64.84% vs. 35.16%) and the mean age of the professionals was 40.5. Two types of health care professionals were included: general physicians (Rural Doctor and General Practitioner) and specialists (Family Medicine Physician and Pediatrician), general physicians were the most frequent health professionals (68.12% vs. 31.88%).

The ICD-10 diagnostic code most frequently assigned by physicians was A09 (Other gastroenteritis and colitis of infectious and unspecified origin) in 98.19% (326/332) of the cases (Table 2).

Table 2. Consultations of acute diarrhea by type of healthcare professional.

**Health professional**	**ICD-10 diagnosis n (%)**				**Total**
	**A06.9**	**A07.1**	**A08.4**	**A09**	
Rural Doctor	0 (0)	0 (0)	0 (0)	57 (17.49)	57
General Practitioner	1 (25)	1 (100)	0 (0)	150 (46.01)	152
Family Medicine Physician	0 (0)	0 (0)	0 (0)	39 (11.96)	39
Pediatrician	3 (75)	0 (0)	1 (100)	80 (24.54)	84
**Total**	**4 (100)**	**1 (100)**	**1 (100)**	**326 (100)**	**332**

A06.9, Amoebiasis, unspecified; A07.1, Giardiasis [lambliasis]; A08.4, Viral intestinal infection, unspecified; A09, Other gastroenteritis and colitis of infectious and unspecified origin.

Resource use

The total resource use in the sample by item is shown in Table 3. A total of 1546 resources were used in 332 children. All children had one primary care contact, and the re-consultation rate was 9.94% (33/332); out of these, just one child needed two re-consultations.

Table 3. Resources used and mean cost per child

Direct sanitary resources	Number of patients	Units dispensed	Mean Cost per child (95%CI)[a]
**Consultations**			
First consultations	332	332	34.49 (34.40 – 34.55)
Re-consultations	33	34	22.88 (21.53 – 24.24)
**Laboratory tests**			
Blood count	38	38	5.10 (5.10 – 5.10)
Urinalysis	22	22	5.69 (5.69 – 5.69)
Gram stain & fresh drop	3	3	2.75 (2.75 – 2.75)
Coprological test	123	123	5.29 (5.29 – 5.29)
WBC, stool	80	80	2.55 (2.55 – 2.55)
Rotavirus	36	36	21.37 (21.37 – 21.37)
FOBT	18	18	3.14 (3.14 – 3.14)
**Prescriptions**			
Antibiotics	88	96	1.75 (1.27 – 2.22)
Antiparasitic	36	57	1.84 (1.65 – 2.02)
Anti-inflammatory	135	136	1.31 (1.25 – 1.37)
Oral rehydration salts	242	553	1.25 (1.18 – 1.33)
Zinc Sulfate	12	18	6.10 (4.59 – 7.61)
**Total cost**	**332**	**1546**	**45.24 (43.71 – 46.76)**

CI, Confident interval; WBC, Fecal leukocyte stain; FOBT, Fecal occult blood test.
[a]Cost presented in International Dollars 2019.

Laboratory tests were performed in 37.95% (126/332) children, representing 320 units of laboratory tests dispensed, of which 38.44% (123/320) were coprological tests, 25% (80/320) fecal leukocyte stain (WBC, stool), 11.25% (36/320) rotavirus tests, 11.88% (38/320) blood count, and 6.88% (22/320) urinalysis (Table 3); in some children, more than one test was ordered, the most frequent combination of tests were coprological test/fecal leukocyte stain in 63.49% (80/126) and coprological test/blood count at 35% (35/126).

Medications were prescribed to 93.67% (311/332) of children. Oral rehydration salts were the most prescribed medication, with 77.81% (242/311), followed by anti-inflammatory drugs with 43.41%

(135/311). Antibiotics were prescribed to 88 children, representing an antibiotic prescription rate of 26.51% (88/332) and antiparasitic were prescribed to 36 children, indicating a prescription rate of 10.84% (36/332). Zinc sulfate was prescribed to only 3.61% (12/332) (Table 3).

A total of 860 units of medication were dispensed, of these oral rehydration salts represented 60.30% (553/860) of the units used, anti-inflammatory drugs 17.79% (136/860), antibiotics 11.16% (96/860), antiparasitic 6.63% (57/860), and zinc sulfate 2.09% (18/860) (Table 3).

Medical contact accounts for 23.67% (366/1546) of the total resource use, prescriptions account for 55.63% (860/1546) and laboratory tests account for 20.70% (320/1546) (Table 4).

Table 4. Total resource use and overall cost of direct sanitary resources.

Direct sanitary resources	Number of patients	Units dispensed	% Resource use	Overall Cost[a]	% Overall Cost[a]
First consultations	332	332	21.47	1,1462.98	76.38
Re-consultations	33	34	2.20	755.29	5.03
Laboratory tests	126	320	20.70	2,007.74	13.38
Prescriptions	311	860	55.63	782.14	5.21
**Total cost**	**332**	**1546**	**100.00**	**15,008.15**	**100.00**

[a]Costs presented in International Dollars 2019.

Cost per episode and overall cost

The mean cost per child per resource used is displayed in Table 3. The mean cost to the MOPH per child per episode of AD was US$45.24 (2019 dollars) (95% CI:43.71, 46.76), the mean cost of laboratory tests was US$15.98 (95% CI: 14.20, 17.76) (2019 dollars), and the mean cost of drugs prescriptions was US$2.51 (95% CI: 2.27, 2.76) (2019 dollars).

The overall cost for the whole sample is presented in Table 4. The total cost of AD for the whole sample was US$15,008.15 (95% CI: 14,511.72, 15,524.32) (2019 dollars). The first consultation accounted for 76.38% of the total cost of the sample and the subsequent consultations accounted for 5.03%. Laboratory tests accounted for 13.38% (US$2,007.74) (2019 dollars) and prescriptions accounted for 5.21% (US$782.14) (2019 dollars) of the overall cost.

The cost by type of laboratory test is shown in Table 5. Rotavirus tests accounted for 38.32% of the overall cost of laboratory tests, follow by coprological test with 32.41%, and white blood cells in stool test with 10.16%.

Table 5. Cost by type of laboratory test.

Type of laboratory test	Units	Total Cost[a]	%
Blood count	38	193.8	9.65
Urinalysis	22	125.18	6.23
Gram stain & fresh drop	3	8.25	0.41
Coprological test	123	650.67	32.41
WBC, stool test	80	204	10.16
Rotavirus	36	769.32	38.32
FOBT	18	56.52	2.82
**Total**	**320**	**2,007.74**	**100**

WBC, stool: Fecal leukocyte stain; FOBT, Fecal occult blood test.
[a]Costs presented in International Dollars 2019.

The cost by type of prescription is reflected in Table 6. Oral rehydration salts accounted for 38.82% of the overall cost of prescriptions, followed by any type of antimicrobial drug (antibiotic/antiparasitic) with 28.13%, analgesics/anti-inflammatory drugs with 22.73%, and zinc sulfate with 10.29%.

Table 6. Cost by type of medication

Type of medication	Units	Total Cost[a]	%
Amoxicillin	14	59.24	7.57

Azithromycin	9	20.94	2.68
Cephalexin	3	7.76	0.99
Clarithromycin	5	34.31	4.39
Trimethoprim/Sulfamethoxazole	65	31.86	4.07
Metronidazole	32	55.84	7.14
Albendazole	25	10.29	1.32
Ibuprofen	9	21.43	2.74
Paracetamol	127	156.37	19.99
Oral rehydration salts	553	303.63	38.82
Zinc Sulfate	18	80.47	10.29
**Total**	**860**	**782.14**	**100**

[a]Costs presented in International Dollars 2019.

Annual Cost to MOPH

We combined the cost per episode results with data on prevalence. The number of consultations for AD in children under five years of age in the ambulatory care centers of the MOPH, according to the National Direction of Statistics and Analysis of the Health Information (DNEAIS), in 2019 was 146,887 [19]. Thus, the annual cost to the MOPH was at least US$6,645,167.88 (2019 dollars) (95% CI: 6,420,430.77 to 6,868,436.12).

Discussion

To the best of our knowledge, this is the first study to measure AD management costs in children under five years of age in Ecuador. Our data provide statistics on resource use and costs from a payer perspective. The cost was estimated from medical records with diagnoses coded according to ICD-10, applying a micro-cost methodology by involving the direct identification and valuation of the resources consumed treating each patient, which improves the accuracy of cost estimation [20].

Medical appointments represent 23.67% of the resources used per AD episode. All children had at least an initial consultation and the rate of re-consultation was almost 10%, which is less than that

reported in the literature [21]. Laboratory tests were ordered in almost 38% of the children, accounting for around the 20% of the resources used.

Medication prescription was the most frequently used resource in the treatment of AD cases. It accounted for 55.63% of all resource units administered. Oral rehydration salts, anti-inflammatory drugs and antibiotics were the most prescribed drugs, 72.89%, 40.66%, and 26.51% respectively; these results are similar to other studies [22–24]. Regardless of this, it is important to mention that the use of resources in medical care may vary according to their availability.

Numerous studies have reported the economic burden of childhood diarrhea in different settings; however, empirical data on the cost of diarrheal illness is sparse [25–29]. We found that the average cost of AD per episode in children under five years of age in Ecuador was US$45.24 (2019 dollars). Our results are similar to those of some countries reported by Rheingans *et al.* [30] for Latin American and Caribbean countries, where total direct medical cost in outpatient settings was US$18.45 in Argentina, US$15.03 in Brazil, 47.25 US$ in Chile, US$30.26 in Honduras, US$33.58 in Mexico, US$39.50 in Panamá and US$30.96 in Venezuela (monetary change to 2019). Nevertheless, the proportion of laboratory and medication costs were higher in Rheingans' study, between 50% and 80%, compared to our data, which was less than 20%. Alternatively, a systematic review of the cost of childhood diarrhea in 137 low- and middle-income countries (LMICs) estimated an average (weighted) US$52.16 per outpatient episode of AD [31], and the direct medical cost represented almost 80% of the total cost, US$41.21 (2020 dollars). These findings are more comparable with our results.

Considerable variation is found in the cost estimates reported in the literature, variation that can be attributed to methodological differences between studies, in particular variation in the perspective

adopted by the studies and the categorization of the costs [32–34]. Additionally, the direct cost associated with treatment could vary according to the capabilities and resources available in different health units, even within the same country, leading to some heterogeneity in the estimated costs of the studies.

The results from our study on the cost of illness may be conservative. As part of the sensitivity analysis, the effect of the proportion of laboratory tests was explored. Otherwise healthy children with AD in ambulatory settings do not require a specific diagnostic work-up, as the results of laboratory investigations are unlikely to change their management [10,35]. According to the Integrated Management of Childhood Illness (IMCI)[36], among other components, to improve the performance of health personnel caring for children under five, it is not necessary to request laboratory tests in outpatient care. However, we found a proportion of laboratory tests ordered of around 13%. If we consider that physicians comply with the recommendations for care according to IMCI, the estimated population cost would be US$ 800,000 lower. Other variables in the study do not greatly affect the results as we found them to be relatively low cost.

Cost-of-illness studies can estimate cost based on epidemiological data, through incidence-based or prevalence-based approaches [37]; the latter is useful for identifying points for cost containment, as this approach provides information on each cost component [38,39].

The total estimated cost of AD in children under five years of age for MOPH in 2019 was about US$6,645,167.88 (2019 dollars) (95% CI: 6,420,430.77 to 6,868,436.12). If laboratory tests had been minimized and antibiotic use cut by at least half, our annual cost estimate would have been reduced by $1 million. Therefore, an intervention that promotes rational use of these resources would be cost saving.

The Integrated Management of Childhood Illness (IMCI) strategy was designed in 1996 by the World Health Organization (WHO) and the United Nations Children's Fund (UNICEF) to improve the quality of care for children in primary care services and was adopted by Ecuador [40]. According to this strategy, in patients under five years of age with AD, it is not necessary to request laboratory tests, and the use of antibiotics is justified in few circumstances; therefore, our findings show that there may be an overuse of these resources and lack of adherence to IMCI recommendations, a situation that has been reported in other studies [41,42]. However, the evaluation of the strategy and its implementation is beyond our research objectives.

A limitation of this study is that it focuses only on the population subsidized by the MOPH; although it covers most of the country's population[43], the results may not be generalizable to other public health subsystems. Another limitation is the perspective used in the study; we did not include patient time costs or other associated resource costs. This perspective is narrower and does not include patients' out-of-pocket costs as they are not borne by payers; therefore, the cost of illness may be underestimated. A strength of the study is that the AD cases were selected from the medical records of different health centers, according to ICD-10, which allowed us to obtain data according to their own diagnoses and thus determine the use of resources for such care. Moreover, the study shows relevant data for decision-making stakeholders to consider cost containment measures for the efficient use of medical resources.

Conclusion:

A high proportion of the direct medical cost of AD in children under five years of age in outpatient settings is due to the unnecessary use of laboratory tests. Factors that may influence the overuse of this resource in primary care need to be explored.

I think it is good to provide a recommendation here to mitigate the observed concerns rather than just asking for more studies.

Acknowledgements:

None

References:

1. Hug L, Sharrow D, Danzhen Y, on behalf of the United Nations Inter-agency Group for Child Mortality Estimation (UN IGME). Levels & Trends in Child Mortality: Report 2019. UNICEF [Internet]. 2019;52. Available from: https://www.unicef.org/media/60561/file/UN-IGME-child-mortality-report-2019.pdf
2. Drancourt M. Acute Diarrhea. In: Infectious Diseases [Internet]. Elsevier; 2017. p. 335-340.e2. Available from: https://linkinghub.elsevier.com/retrieve/pii/B9780702062858000381
3. Radlovic N, Lekovic Z, Vuletic B, Radlovic V, Simic D. Acute diarrhea in children. Srp Arh Celok Lek [Internet]. 2015;143(11–12):755–62. Available from: http://www.doiserbia.nb.rs/Article.aspx?ID=0370-81791512755R
4. Pinzón-Rondón ÁM, Zárate-Ardila C, Hoyos-Martínez A, Ruiz-Sternberg ÁM, Vélez-van-Meerbeke A. Country characteristics and acute diarrhea in children from developing nations: a multilevel study. BMC Public Health [Internet]. 2015 Dec 21;15(1):811. Available from: http://bmcpublichealth.biomedcentral.com/articles/10.1186/s12889-015-2120-8
5. Troeger C, Forouzanfar M, Rao PC, Khalil I, Brown A, Reiner RC, et al. Estimates of global, regional, and national morbidity, mortality, and aetiologies of diarrhoeal diseases: a systematic analysis for the Global Burden of Disease Study 2015. Lancet Infect Dis [Internet]. 2017 Sep;17(9):909–48. Available from:

https://linkinghub.elsevier.com/retrieve/pii/S1473309917302761

6. Cheng AC, McDonald JR, Thielman NM. Infectious Diarrhea in Developed and Developing Countries. J Clin Gastroenterol [Internet]. 2005 Oct;39(9):757–73. Available from: http://journals.lww.com/00004836-200510000-00002
7. Aranda-Michel J, Giannella RA. Acute diarrhea: a practical review. Am J Med [Internet]. 1999 Jun;106(6):670–6. Available from: https://linkinghub.elsevier.com/retrieve/pii/S000293439900128X
8. Florez ID, Veroniki A-A, Al Khalifah R, Yepes-Nuñez JJ, Sierra JM, Vernooij RWM, et al. Comparative effectiveness and safety of interventions for acute diarrhea and gastroenteritis in children: A systematic review and network meta-analysis. van Wouwe JP, editor. PLoS One [Internet]. 2018 Dec 5;13(12):e0207701. Available from: https://dx.plos.org/10.1371/journal.pone.0207701
9. Lo Vecchio A, Dias JA, Berkley JA, Boey C, Cohen MB, Cruchet S, et al. Comparison of Recommendations in Clinical Practice Guidelines for Acute Gastroenteritis in Children. J Pediatr Gastroenterol Nutr [Internet]. 2016 Aug;63(2):226–35. Available from: https://journals.lww.com/00005176-201608000-00011
10. Guarino A, Lo Vecchio A, Dias JA, Berkley JA, Boey C, Bruzzese D, et al. Universal Recommendations for the Management of Acute Diarrhea in Nonmalnourished Children. J Pediatr Gastroenterol Nutr [Internet]. 2018 Nov;67(5):586–93. Available from: https://journals.lww.com/00005176-201811000-00011
11. Carter E, Bryce J, Perin J, Newby H. Harmful practices in the management of childhood diarrhea in low- and middle-income countries: a systematic review. BMC Public Health [Internet]. 2015 Dec 18;15(1):788. Available from: http://bmcpublichealth.biomedcentral.com/articles/10.1186/s12889-015-2127-1

12. Brownlee S, Chalkidou K, Doust J, Elshaug AG, Glasziou P, Heath I, et al. Evidence for overuse of medical services around the world. Lancet [Internet]. 2017 Jul;390(10090):156–68. Available from: https://linkinghub.elsevier.com/retrieve/pii/S0140673616325855

13. Ministerio de Salud Pública del Ecuador. Información Estadística y Geográfica de Salud [Internet]. 2017 [cited 2020 Jun 7]. Available from: https://www.salud.gob.ec/informacion-estadistica-de-produccion-de-salud/#

14. Lucio R, Villacrés N, Henríquez R. Sistema de salud de Ecuador. Salud Publica Mex [Internet]. 2011 [cited 2015 Jan 15];53(1):177–87. Available from: http://www.scielo.org.mx/scielo.php?pid=S0036-36342011000800013&script=sci_arttext

15. Instituto Nacional de Estadísticas y Censos (INEC-Ecuador). Encuesta de Condiciones de Vida. ECV [Internet]. 2015. Available from: https://www.ecuadorencifras.gob.ec/encuesta-de-condiciones-de-vida-ecv

16. Ministerio de Salud Pública del Ecuador. Manual del Modelo de Atencion Integral del Sistema Nacional de Salud Familiar Comunitario e Intercultural (MAIS-FCI) [Internet]. 2012 [cited 2019 Jun 28]. Available from: http://instituciones.msp.gob.ec/somossalud/images/documentos/guia/Manual_MAIS-MSP12.12.12.pdf

17. International Monetary Fund. World Economic Outlook Database. 2017; Available from: https://www.imf.org/external/pubs/ft/weo/2017/02/weodata/index.aspx

18. Ministerio de Salud Pública del Ecuador. Tarifario de Prestaciones para el Sistema Nacional de Salud [Internet]. 2014. Available from: https://aplicaciones.msp.gob.ec/salud/archivosdigitales/documentosDirecciones/dnn/archivos/TARIFARIO.pdf

19. Dirección Nacional de Estadística y Análisis de Información de Salud – Ministerio de Salud Pública. Registro Diario Automatizado de Consultas y Atenciones Ambulatorias (RDACAA 1.6 y RDACAA 2.0) y Plataforma de registro en atenciones de salud (PRAS) [Internet]. 2019 [cited 2022 Apr 21]. Available from: https://www.salud.gob.ec/direccion-nacional-de-estadistica-y-analisis-de-informacion-de-salud/

20. Xu X, Grossetta Nardini HK, Ruger JP. Micro-costing studies in the health and medical literature: protocol for a systematic review. Syst Rev [Internet]. 2014 Dec 21;3(1):47. Available from: https://systematicreviewsjournal.biomedcentral.com/articles/10.1186/2046-4053-3-47

21. Rautenberg TA. Evaluating the cost utility of racecadotril for the treatment of acute watery diarrhea in children: the RAWD model. Clin Outcomes Res [Internet]. 2012 Apr;109. Available from: http://www.dovepress.com/evaluating-the-cost-utility-of-racecadotril-for-the-treatment-of-acute-peer-reviewed-article-CEOR

22. Okubo Y, Miyairi I, Michihata N, Morisaki N, Kinoshita N, Urayama KY, et al. Recent Prescription Patterns for Children With Acute Infectious Diarrhea. J Pediatr Gastroenterol Nutr [Internet]. 2019 Jan;68(1):13–6. Available from: https://journals.lww.com/00005176-201901000-00004

23. Efunshile AM, Ezeanosike O, Nwangwu CC, König B, Jokelainen P, Robertson LJ. Apparent overuse of antibiotics in the management of watery diarrhoea in children in Abakaliki, Nigeria. BMC Infect Dis [Internet]. 2019 Dec 21;19(1):275. Available from: https://bmcinfectdis.biomedcentral.com/articles/10.1186/s12879-019-3899-1

24. Castellano VE, Giglio ND, Pacchiotti AC, Gentile Á. Outpatient management of childhood acute diarrhea: survey among pediatricians from a children's hospital in the City of Buenos Aires. Arch Argent Pediatr [Internet]. 2022 Feb 1;120(1):46–53. Available

from: https://www.sap.org.ar/docs/publicaciones/archivosarg/2022/v120n1a07e.pdf
25. Ehrenkranz P, Lanata CF, Penny ME, Salazar-Lindo E, Glass RI. Rotavirus diarrhea disease burden in Peru: the need for a rotavirus vaccine and its potential cost savings. Rev Panam Salud Pública [Internet]. 2001 Oct;10(4):240–8. Available from: http://www.scielosp.org/scielo.php?script=sci_arttext&pid=S1020-49892001001000004&lng=en&nrm=iso&tlng=en
26. Rochanathimoke O, Riewpaiboon A, Tharmaphornpilas P, Jiamsiri S, Thavorncharoensap M, Postma MJ. Economic burden of rotavirus diarrhea in Thailand: Report from a pilot study on rotavirus vaccination. Vaccine [Internet]. 2019 Jan;37(4):587–94. Available from: https://linkinghub.elsevier.com/retrieve/pii/S0264410X18316736
27. Ngabo F, Mvundura M, Gazley L, Gatera M, Rugambwa C, Kayonga E, et al. The Economic Burden Attributable to a Child's Inpatient Admission for Diarrheal Disease in Rwanda. Griffiths UK, editor. PLoS One [Internet]. 2016 Feb 22;11(2):e0149805. Available from: https://dx.plos.org/10.1371/journal.pone.0149805
28. Hasan MZ, Mehdi GG, De Broucker G, Ahmed S, Ali MW, Martin Del Campo J, et al. The economic burden of diarrhea in children under 5 years in Bangladesh. Int J Infect Dis [Internet]. 2021 Jun;107:37–46. Available from: https://linkinghub.elsevier.com/retrieve/pii/S1201971221003490
29. Rheingans R, Anderson JD, Bagamian KH, Laytner LA, Pecenka CJ, Gilani SSA, et al. Effects of geographic and economic heterogeneity on the burden of rotavirus diarrhea and the impact and cost-effectiveness of vaccination in Pakistan. Vaccine [Internet]. 2018 Dec;36(51):7780–9. Available from: https://linkinghub.elsevier.com/retrieve/pii/S0264410X18301725
30. Rheingans RD, Constenla D, Antil L, Innis BL, Breuer T. Economic and health burden of

rotavirus gastroenteritis for the 2003 birth cohort in eight Latin American and Caribbean countries. Rev Panam Salud Publica/Pan Am J Public Heal. 2007;21(4):192–204.

31. Baral R, Nonvignon J, Debellut F, Agyemang SA, Clark A, Pecenka C. Cost of illness for childhood diarrhea in low- And middle-income countries: A systematic review of evidence and modelled estimates. BMC Public Health. 2020;20(1):1–13.

32. Alvis-Guzman N, Orozco-Africano J, Paternina-Caicedo A, Coronell-Rodríguez W, Alvis-Estrada L, Jervis-Jálabe D, et al. Treatment costs of diarrheal disease and all-cause pneumonia among children under-5 years of age in Colombia. Vaccine [Internet]. 2013 Jul;31:C58–62. Available from: https://linkinghub.elsevier.com/retrieve/pii/S0264410X13006130

33. Burke RM, Smith ER, Dahl RM, Rebolledo PA, del Carmen Calderón M, Cañipa B, et al. The economic burden of pediatric gastroenteritis to Bolivian families: a cross-sectional study of correlates of catastrophic cost and overall cost burden. BMC Public Health [Internet]. 2014 Dec 24;14(1):642. Available from: http://bmcpublichealth.biomedcentral.com/articles/10.1186/1471-2458-14-642

34. Burke RM, Rebolledo PA, Embrey SR, Wagner LD, Cowden CL, Kelly FM, et al. The burden of pediatric diarrhea: a cross-sectional study of incurred costs and perceptions of cost among Bolivian families. BMC Public Health [Internet]. 2013 Dec 2;13(1):708. Available from: https://bmcpublichealth.biomedcentral.com/articles/10.1186/1471-2458-13-708

35. Florez ID, Niño-Serna LF, Beltrán-Arroyave CP. Acute Infectious Diarrhea and Gastroenteritis in Children. Curr Infect Dis Rep [Internet]. 2020 Feb 28;22(2):4. Available from: http://link.springer.com/10.1007/s11908-020-0713-6

36. Ministerio de Salud Pública del Ecudor - Dirección Nacional de Normatización. Atención

Integrada a Enfermedades Prevalentes de la Infancia (AIEPI) Clínico. Cuadro de Procedimientos. Actualización 2017 [Internet]. Quito; 2017. 92 p. Available from: http://instituciones.msp.gob.ec/images/Documentos/AIEPI_6feb_2019_web.pdf

37. Jo C. Cost-of-illness studies: concepts, scopes, and methods. Clin Mol Hepatol [Internet]. 2014;20(4):327. Available from: http://e-cmh.org/journal/view.php?doi=10.3350/cmh.2014.20.4.327

38. Tarricone R. Cost-of-illness analysis. Health Policy (New York) [Internet]. 2006 Jun;77(1):51–63. Available from: https://linkinghub.elsevier.com/retrieve/pii/S0168851005001867

39. Sánchez Choez X, Loaiza Martínez M, Vaca Tatamuez V, López Peña M, Manzano Pasquel A, Jimbo Sotomayor R. Medical Cost of Upper Respiratory Tract Infections in Children in Ambulatory Care. Value Heal Reg Issues [Internet]. 2021;26:1–9. Available from: http://dx.doi.org/10.1016/j.vhri.2020.10.001

40. Lambrechts T, Bryce J, Orinda V. Integrated management of childhood illness: a summary of first experiences. Bull World Health Organ [Internet]. 1999;77(7):582–94. Available from: http://www.ncbi.nlm.nih.gov/pubmed/10444882

41. Oliphant NP, Manda S, Daniels K, Odendaal WA, Besada D, Kinney M, et al. Integrated community case management of childhood illness in low- and middle-income countries. Cochrane Database Syst Rev [Internet]. 2021 Feb 10;2021(2). Available from: http://doi.wiley.com/10.1002/14651858.CD012882.pub2

42. Gera T, Shah D, Garner P, Richardson M, Sachdev HS. Integrated management of childhood illness (IMCI) strategy for children under five. Cochrane Database Syst Rev [Internet]. 2016 Jun 22; Available from: https://doi.wiley.com/10.1002/14651858.CD010123.pub2

43. Ministerio de Salud Pública del Ecuador. En 2018: se evidenció un Sistema Nacional de Salud fortalecido – Ministerio de Salud Pública [Internet]. 2019 [cited 2022 Jun 3]. Available from: https://www.salud.gob.ec/en-2018-se-evidencio-un-sistema-nacional-de-salud-fortalecido/

Supporting information

S1 Table. Cases registered as acute diarrhea in the pediatric population under five years of age

S2 Table. Source and value of unit costs

Point by point response letter

Academic Editor

"1. Please ensure that your manuscript meets PLOS ONE's style requirements, including those for file naming. The PLOS ONE style templates can be found at https://journals.plos.org/plosone/s/file?id=wjVg/PLOSOne_formatting_sample_main_body.pdf and https://journals.plos.org/plosone/s/file?id=ba62/PLOSOne_formatting_sample_title_authors_affiliations.pdf"

Corrections were made to meet PLOS ONE's style requirements.

"2. Please update your submission to use the PLOS LaTeX template. The template and more information on our requirements for LaTeX submissions can be found at http://journals.plos.org/plosone/s/latex."

PLOS template was used in this submission.

"3. Please include your full ethics statement in the 'Methods' section of your manuscript file. In your statement, please include the full name of the IRB or ethics committee who approved or waived your study, as well as whether or not you obtained informed written or verbal consent. If consent was waived for your study, please include this information in your statement as well."

Full ethics statement was included in methods section. Lines 78-81

Reviewer #1:

"1. The whole manuscript should be revised for improved English and grammatical mistakes."

Suggestion accepted. The manuscript has been proofread by a native speaker.

"2. Line 38, %% should be corrected to %, similarly E.coli should be written as E.Coli. All bacterial pathogens name should be written in italic form."

Suggestion accepted. Correction was made. Lines 52-53

"3. Line 79, 'The district is distributed in 21 ambulatory medical care centres, These health centres are distributed in rural and urban areas of the city and offer health services to 85% of the population'. According to WHO criteria, 30 prescription per facility should be analysed. So the sample size should be at least 630."

Observational burden of illness studies are widely used to characterize treatment patterns, resource utilization and costs associated with a disease. For a burden of illness study the aim of sample size calculation is to ensure sufficient precision in descriptive outcomes, e.g. characterized by the width of 95% confidence intervals (CIs). We followed sampling guidelines in cost-of-illness studies designed for practical application in real-world studies (1,2). The sampling approach used in our study complies with the methodological recommendations according to the guidelines for conducting health economics studies.

1. Johnston KM, Lakzadeh P, Donato BMK, Szabo SM. Methods of sample size calculation in descriptive retrospective burden of illness studies. BMC Med Res Methodol [Internet]. 2019;19(1):9. Available from: http://www.ncbi.nlm.nih.gov/pubmed/30626343
2. 1. Jo C. Cost-of-illness studies: concepts, scopes, and methods. Clin Mol Hepatol [Internet]. 2014;20(4):327. Available from: http://e-cmh.org/journal/view.php?doi=10.3350/cmh.2014.20.4.327

"4. Line 123, Estimation of annual cost is not clear. How total number of AD episodes per year of the same patient was calculated?"

The annual cost was estimated as cost per episode per child in 2019 × total number of episodes in 2019. As this is a cost-of-illness study, all costs are imputed to each child to estimate an average cost. If the same child has had several episodes of the disease, this is already considered in the estimate. The methodology (1,2,3) for estimating disease costs provides an estimate of the burden resulting from the prevalence of the disease over a given period, most often a year. This is called the prevalence approach.

1. Hodgson TA, Meiners MR. Cost-of-Illness Methodology: A Guide to Current Practices and Procedures. Milbank Mem Fund Q Health Soc. 1982;60(3):429. Available from: https://www.jstor.org/stable/3349801?origin=crossref
2. Drummond MF: Methods for the economic evaluation of health care programmes. 3rd edition. Oxford: Oxford University Press; 2005.
3. Jo C. Cost-of-illness studies: concepts, scopes, and methods. Clin Mol Hepatol [Internet]. 2014;20(4):327. Available from: http://e-cmh.org/journal/view.php?doi=10.3350/cmh.2014.20.4.327

"5. Name of health facilities should be mentioned in a tabular form, mentioning the number of samples taken per facility."

Suggestion accepted. Data is displayed in a Supplementary Table S1.

"6. I could not find the standard treatment of AD given by WHO. Please correlate your study with STGs of AD with WHO guidelines. You can add another variable of Adherence to STGs in your study. For your ease please read

and cite the following article.
https://dx.doi.org/10.21608/bfsa.2020.93568”

The standard treatment of acute diarrhea is cited. A cost-of-illness study, such as this one, focuses on measuring the amount of resources used for a given disease and estimating the monetary expenditure. Therefore, a variable such *as "adherence to standard treatment regimen"* is not necessary (it is not a resource used). The implications derived from this variable go beyond our primary objective.

“7. et al should be italic.”

Suggestion accepted.

“8. Conclusion should be rewritten. As lab test is necessary so I don’t think that treatment cost is increased due to unnecessary testing. So revise conclusion according to prescribed medicines and its cost based on local or multinational brands.”

We politely disagree with the reviewer. According to the guidelines for treating acute diarrhea in children, laboratory testing is not necessary in the outpatient setting, as it will not modify the treatment. Therefore, the use of this resource reflects an unnecessary cost.

“9. No statistical test was applied on the variables.”

A cost-of-illness study, such as this one, focuses on measuring the amount of resources used for a given disease and estimating the monetary expenditure. Advanced statistical tests are not necessary.

“Other comments:
The article focuses on treatment cost which is divided into two types, 1. Medication cost and 2. Lab test cost. On the other hand the article describes number of units dispensed from the facilities. So these two parameters should be clearly separated by headings and discussed accordingly in separate results heading.”

Suggestion accepted. Results were better discussed following the recommendation but meeting PLOS ONE’s style requirements

---

Reviewer #2:

“Authors should be more clear on the methodology and presentation of results in approach that can be understood by a layman. The comments provided in the attached pdf documents should be addressed clearly and correctly.”

Suggestion accepted. This manuscript version has been corrected and rewritten to facilitate comprehension.

All comments provided were properly addressed.

1. A definition of international dollar has been added. Lines 130-131.
2. Table 1 now includes information on comorbidities found in the sample. Line 152
3. Table 3 was reworked to improve comprehension. Line 176
4. US$ is used in the manuscript as the standard unit.

"This part is not clear and confusing. The prevalence rate is not the same as the sample size. I disagree that you can extrapolate the data on national prevalence to estimate annual cost. The annual cost should stay within your sample size. This is because health cost will vary by location. So, you can not assume the cost in your location is the same as elsewhere."

Cost-of-illness studies can be described as prevalence-based or incidence-based approaches, depending on how the epidemiological data are used. Prevalence-based studies can use this procedure to extrapolate cost and resource data over a period of time (1,2). This is called a prevalence approach. We partially agree with the reviewer, as this type of estimation may have some limitations; however, this is discussed in lines 260-655. For this reason, it is advisable to perform a sensitivity analysis (lines 266-276).

1. Hodgson TA, Meiners MR. Cost-of-Illness Methodology: A Guide to Current Practices and Procedures. Milbank Mem Fund Q Health Soc. 1982;60(3):429. Available from: https://www.jstor.org/stable/3349801?origin=crossref
2. Jo C. Cost-of-illness studies: concepts, scopes, and methods. Clin Mol Hepatol [Internet]. 2014;20(4):327. Available from: http://e-cmh.org/journal/view.php?doi=10.3350/cmh.2014.20.4.327

"The term sensitivity analysis is not appropriate here. The information provided here can be included in the discussion but not discussed as sensitivity analysis"

Suggestion accepted. We discussed this analysis in the proper section. Lines 265-274.

"add " to the best of our knowledge""
"Remove the word reliable as there is no way your audience can confirm that adjective. It is ok to write that you provided data on resources used but you don't need to stress the reliability. The reliability of resources may be difficult to measure in this context."

Suggestions accepted. Lines 231-233

"If you say numerous studies, you should cite references backing that statement."

Suggestion accepted. Lines 246-247

"Rather say the result may not be generalized since it is a limitation and not that it can be generalized."

Suggestion accepted. We have rephrased the sentence to improve comprehension. Lines 291-293.
