# Supplementary material for: Medical cost of acute diarrhea in children in ambulatory care
Source: PLoS One. 2022 Dec 16;17(12):e0279239. doi: 10.1371/journal.pone.0279239 (PMC9757569; doi:10.1371/journal.pone.0279239)
Supplement: Attachment — Submitted filename: Response to Reviewers v1.docx [file pone.0279239.s004.docx]

***Point by point response letter***

***Academic Editor***

***“1. Please ensure that your manuscript meets PLOS ONE's style requirements, including those for file naming. The PLOS ONE style templates can be found at 
https://journals.plos.org/plosone/s/file?id=wjVg/PLOSOne_formatting_sample_main_body.pdf and 
https://journals.plos.org/plosone/s/file?id=ba62/PLOSOne_formatting_sample_title_authors_affiliations.pdf”***

Corrections were made to meet PLOS ONE’s style requirements.

***“2. Please update your submission to use the PLOS LaTeX template. The template and more information on our requirements for LaTeX submissions can be found at*** [***http://journals.plos.org/plosone/s/latex***](http://journals.plos.org/plosone/s/latex)***.”***

*PLOS template was used in this submission.*

***“3. Please include your full ethics statement in the ‘Methods’ section of your manuscript file. In your statement, please include the full name of the IRB or ethics committee who approved or waived your study, as well as whether or not you obtained informed written or verbal consent. If consent was waived for your study, please include this information in your statement as well.”***

Full ethics statement was included in methods section. Lines 78-81

***Reviewer #1:***

***“1. The whole manuscript should be revised for improved English and grammatical mistakes.”***

Suggestion accepted. The manuscript has been proofread by a native speaker.

***“2. Line 38, %% should be corrected to %, similarly E.coli should be written as E.Coli. All bacterial pathogens name should be written in italic form.”***

Suggestion accepted. Correction was made. Lines 52-53

***“3. Line 79, ‘The district is distributed in 21 ambulatory medical care centres, These health centres are distributed in rural and urban areas of the city and offer health services to 85% of the population’. According to WHO criteria, 30 prescription per facility should be analysed. So the sample size should be at least 630.”***

Observational burden of illness studies are widely used to characterize treatment patterns, resource utilization and costs associated with a disease. For a burden of illness study the aim of sample size calculation is to ensure sufficient precision in descriptive outcomes, e.g. characterized by the width of 95% confidence intervals (CIs). We followed sampling guidelines in cost-of-illness studies designed for practical application in real-world studies (1,2). The sampling approach used in our study complies with the methodological recommendations according to the guidelines for conducting health economics studies.

1. Johnston KM, Lakzadeh P, Donato BMK, Szabo SM. Methods of sample size calculation in descriptive retrospective burden of illness studies. BMC Med Res Methodol [Internet]. 2019;19(1):9. Available from: http://www.ncbi.nlm.nih.gov/pubmed/30626343
2. 1. Jo C. Cost-of-illness studies: concepts, scopes, and methods. Clin Mol Hepatol [Internet]. 2014;20(4):327. Available from: http://e-cmh.org/journal/view.php?doi=10.3350/cmh.2014.20.4.327

***“4. Line 123, Estimation of annual cost is not clear. How total number of AD episodes per year of the same patient was calculated?”***

The annual cost was estimated as cost per episode per child in 2019 × total number of episodes in 2019. As this is a cost-of-illness study, all costs are imputed to each child to estimate an average cost. If the same child has had several episodes of the disease, this is already considered in the estimate. The methodology (1,2,3) for estimating disease costs provides an estimate of the burden resulting from the prevalence of the disease over a given period, most often a year. This is called the prevalence approach.

1. Hodgson TA, Meiners MR. Cost-of-Illness Methodology: A Guide to Current Practices and Procedures. Milbank Mem Fund Q Health Soc. 1982;60(3):429. Available from: <https://www.jstor.org/stable/3349801?origin=crossref>
2. Drummond MF: Methods for the economic evaluation of health care programmes. 3rd edition. Oxford: Oxford University Press; 2005.
3. Jo C. Cost-of-illness studies: concepts, scopes, and methods. Clin Mol Hepatol [Internet]. 2014;20(4):327. Available from: http://e-cmh.org/journal/view.php?doi=10.3350/cmh.2014.20.4.327

***“5. Name of health facilities should be mentioned in a tabular form, mentioning the number of samples taken per facility.”***

Suggestion accepted. Data is displayed in a Supplementary Table S1.

***“6. I could not find the standard treatment of AD given by WHO. Please correlate your study with STGs of AD with WHO guidelines. You can add another variable of Adherence to STGs in your study. For your ease please read and cite the following article.***
[***https://dx.doi.org/10.21608/bfsa.2020.93568***](https://dx.doi.org/10.21608/bfsa.2020.93568)***”***

The standard treatment of acute diarrhea is cited. A cost-of-illness study, such as this one, focuses on measuring the amount of resources used for a given disease and estimating the monetary expenditure. Therefore, a variable such *as "adherence to standard treatment regimen"* is not necessary (it is not a resource used). The implications derived from this variable go beyond our primary objective.

***“7. et al should be italic.”***

Suggestion accepted.

***“8. Conclusion should be rewritten. As lab test is necessary so I don’t think that treatment cost is increased due to unnecessary testing. So revise conclusion according to prescribed medicines and its cost based on local or multinational brands.”***

*We politely disagree with the reviewer. According to the guidelines for treating acute diarrhea in children, laboratory testing is not necessary in the outpatient setting, as it will not modify the treatment. Therefore, the use of this resource reflects an unnecessary cost.*

***“9. No statistical test was applied on the variables.”***

A cost-of-illness study, such as this one, focuses on measuring the amount of resources used for a given disease and estimating the monetary expenditure. Advanced statistical tests are not necessary.

***“Other comments:
The article focuses on treatment cost which is divided into two types, 1. Medication cost and 2. Lab test cost. On the other hand the article describes number of units dispensed from the facilities. So these two parameters should be clearly separated by headings and discussed accordingly in separate results heading.”***

Suggestion accepted. Results were better discussed following the recommendation but meeting PLOS ONE’s style requirements

***Reviewer #2:***

***“Authors should be more clear on the methodology and presentation of results in approach that can be understood by a layman. The comments provided in the attached pdf documents should be addressed clearly and correctly.”***

Suggestion accepted. This manuscript version has been corrected and rewritten to facilitate comprehension.

All comments provided were properly addressed.

1. A definition of international dollar has been added. Lines 130-131.
2. Table 1 now includes information on comorbidities found in the sample. Line 152
3. Table 3 was reworked to improve comprehension. Line 176
4. US$ is used in the manuscript as the standard unit.

***“This part is not clear and confusing. The prevalence rate is not the same as the sample size. I disagree that you can extrapolate the data on national prevalence to estimate annual cost. The annual cost should stay within your sample size. This is because health cost will vary by location. So, you can not assume the cost in your location is the same as elsewhere.”***

*Cost-of-illness studies can be described as prevalence-based or incidence-based approaches, depending on how the epidemiological data are used. Prevalence-based studies can use this procedure to extrapolate cost and resource data over a period of time (1,2). This is called a prevalence approach. We partially agree with the reviewer, as this type of estimation may have some limitations; however, this is discussed in lines 260-655. For this reason, it is advisable to perform a sensitivity analysis (lines 266-276).*

1. Hodgson TA, Meiners MR. Cost-of-Illness Methodology: A Guide to Current Practices and Procedures. Milbank Mem Fund Q Health Soc. 1982;60(3):429. Available from: https://www.jstor.org/stable/3349801?origin=crossref
2. Jo C. Cost-of-illness studies: concepts, scopes, and methods. Clin Mol Hepatol [Internet]. 2014;20(4):327. Available from: http://e-cmh.org/journal/view.php?doi=10.3350/cmh.2014.20.4.327

***“The term sensitivity analysis is not appropriate here. The information provided here can be included in the discussion but not discussed as sensitivity analysis”***

Suggestion accepted. We discussed this analysis in the proper section. Lines 265-274.

***“add " to the best of our knowledge"”***

***“Remove the word reliable as there is no way your audience can confirm that adjective. It is ok to write that you provided data on resources used but you don't need to stress the reliability. The reliability of resources may be difficult to measure in this context.”***

Suggestions accepted. Lines 231-233

***“If you say numerous studies, you should cite references backing that statement.”***

Suggestion accepted. Lines 246-247

***“Rather say the result may not be generalized since it is a limitation and not that it can be generalized.”***

Suggestion accepted. We have rephrased the sentence to improve comprehension. Lines 291-293.
