# Supplementary material for: Medical cost of acute diarrhea in children in ambulatory care
Source: PLoS One. 2022 Dec 16;17(12):e0279239. doi: 10.1371/journal.pone.0279239 (PMC9757569; doi:10.1371/journal.pone.0279239)
Supplement: Attachment — Submitted filename: Response to Reviewers v2.docx [file pone.0279239.s007.docx]

***Point by point response letter***

***Journal requirements***

***Please review your reference list to ensure that it is complete and correct. If you have cited papers that have been retracted, please include the rationale for doing so in the manuscript text, or remove these references and replace them with relevant current references. Any changes to the reference list should be mentioned in the rebuttal letter that accompanies your revised manuscript. If you need to cite a retracted article, indicate the article’s retracted status in the References list and also include a citation and full reference for the retraction notice.***

Corrections were made to meet PLOS ONE’s journal requirements. Reference number 12 was changed. Brownlee, 2017. (With an erratum) by Elshaug, 2017. Line 340.

***Reviewer #1:***

***“The manuscript is improved as compared to last version. Authors have done a good job. English is highly improved.”***

No changes were made.

***Reviewer #2:***

***“I think the authors have done justice to the earlier queries. However, the conclusion needs a slight modification. Rather than providing a need for more research, it might be nice to offer recommendation to mitigate the challenges identified from the study in the conclusion. I know that some recommendations were in the body of the discussion, but it would be nice to input a summarized version of the recommendation in the conclusion in a concise manner.***

Corrections were made in the conclusion section. Lines 300-304.
