# Supplementary material for: Medical cost of acute diarrhea in children in ambulatory care
Source: PLoS One. 2022 Dec 16;17(12):e0279239. doi: 10.1371/journal.pone.0279239 (PMC9757569; doi:10.1371/journal.pone.0279239)
Supplement: Attachment — Submitted filename: Plos one reviewer comments by Azeem (Autosaved).docx [file pone.0279239.s006.docx]

**Other comments:**

Good job done by authors.
